# Antisense oligonucleotides targeting valosin‐containing protein ameliorate muscle pathology and molecular defects in cell and mouse models of multisystem proteinopathy

**DOI:** 10.1002/ctm2.70530

**Published:** 2025-12-08

**Authors:** Pallabi Pal, Michele Carrer, Lan Weiss, Olga G. Jaime, Cheng Cheng, Alyaa Shmara, Victoria Boock, Danae Bosch, Marwan Youssef, Yasamin Fazeli, Megan Afetian, Tamar R. Grossman, Michael R. Hicks, Paymaan Jafar‐nejad, Virginia Kimonis

**Affiliations:** ^1^ Department of Pediatrics Division of Genetics and Genomic Medicine University of California Irvine California USA; ^2^ Ionis Pharmaceuticals, Inc. Carlsbad California USA; ^3^ Department of Physiology and Biophysics School of Medicine University of California Irvine California USA; ^4^ Sue and Bill Gross Stem Cell Research Center University of California Irvine California USA; ^5^ Present address: La Jolla Labs El Cajon CA 92020 USA

**Keywords:** A232E mouse model, induced pluripotent stem cell (IPSC), skeletal muscle progenitor cell (SMPC), VCP R155H cell model

## Abstract

**Background:**

Valosin‐containing protein (VCP) related disease, also known as multisystem proteinopathy 1 (MSP1), is an autosomal dominant disease caused by gain‐of‐function pathogenic variants of the *VCP* gene. The disease presents with variable combinations of inclusion body myopathy, early‐onset Paget's disease of bone, frontotemporal dementia and may also overlap with familial amyotrophic lateral sclerosis. There is currently no treatment for this progressive disease associated with early demise resulting from proximal limb girdle and respiratory muscle weakness. We hypothesise that regulating VCP hyperactivity to normal levels can reduce the disease pathology.

**Main topics covered:**

In this study, we assessed the effect of antisense oligonucleotides (ASOs) specifically targeting the human *VCP* gene in the patient (R155H) iPSC‐derived skeletal muscle progenitor cells (SMPCs). ASOs were well tolerated up to a concentration of 5 µM and significantly reduced VCP protein expression in the SMPCs by 48% (95% CI [39–56]). We also treated the transgenic mouse model of VCP disease with the overexpressed humanised VCP severe A232E pathogenic gene variant (VCP A232E mice) with weekly subcutaneous ASO injections starting from 6 months of age for 3 months. In the skeletal muscle of transgenic mice, ASOs resulted in 30% (95% CI [27–32]) knockdown of VCP protein compared with control ASO. The ASO‐mediated reduction of VCP expression in muscle tissue was associated with improvement in autophagy flux and reduction in TAR DNA binding protein 43 (TDP‐43) expression, hallmarks of VCP related MSP1. In addition, ASO‐treated VCP A232E mice showed improvements in functional tests of muscle strength, such as rotarod and inverted screen test compared with mice treated with control ASO.

**Conclusions:**

These results suggest that targeting VCP could be beneficial in preventing the progression of the VCP myopathy and hold promise for the treatment of patients with VCP related MSP1.

**Key points:**

1. VCP multisystem proteinopathy 1 is caused by gain‐of‐function pathogenic variants of the VCP gene.
2. VCP targeting ASOs were well tolerated and significantly reduced VCP, TAR DNA binding protein 43 (TDP 43), and autophagy protein expression in the (R155H) iPSC‐derived skeletal muscle progenitor cells (SMPCs).
3. The ASOs reduced VCP, TDP‐43, and autophagy flux expression, and improved functional tests of muscle strength in the humanized VCP A232E mice.

## Graphical abstract

Multisystem proteinopathy 1 (MSP1), caused by gain‐of‐function VCP variants, leads to multisystem degeneration. Using VCP patient‐derived hiPSCs, skeletal muscle progenitor cells were generated to evaluate antisense oligonucleotide (ASO) therapy. ASOs reduced VCP expression, restored autophagy and decreased TDP‐43 pathology in patient cells and humanized transgenic mice, supporting ASO‐mediated RNA modulation as a promising MSP1 treatment.

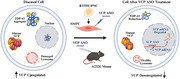

## 1 INTRODUCTION

Multisystem proteinopathy 1 (MSP1) or inclusion body myopathy (IBM) associated with Paget's disease of the bone, frontotemporal dementia (FTD) (IBMPFD), and amyotrophic lateral sclerosis (ALS) is a rare syndromic disease caused by gain‐of‐function variants in the valosin‐containing protein (*VCP*) gene.^1^, ^2^ Over 85 pathogenic variants have been identified in more than 400 patients worldwide, 80–90% of whom have myopathy predominantly affecting the proximal girdle muscle, the diaphragm and other muscle groups.^3^ Paget disease of bone is present in 49% of patients and affects the pelvis, skull, scapulae and vertebral column, resulting in pain, bone deformities and fractures.^4^, ^5^ Additionally, 27% of patients have premature frontotemporal dementia and manifest signs of neurodegeneration in the frontal and temporal lobes, including dysnomia, comprehension deficits and social unawareness, and approximately 15% of patients have ALS.^6^, ^7^ There are currently no effective treatments available for the neuromuscular components of MSP1, and patients generally die from respiratory failure and cardiomyopathy, typically in their 50s to 60s. Therefore, development of a treatment for this devastatingly progressive VCP‐related neuromuscular disease is urgently needed.

VCP is a ubiquitously expressed hexameric protein belonging to the ATPases associated with various activities (AAA+) chaperone‐like protein family. VCP is a critically important component of the ubiquitin–proteasome system. Mutation in one VCP allele can lead to a wide array of dysfunctions, including improper protein degradation and a lack of protein quality control. Cellular responses to VCP mutant range from aberrant cell cycle regulation, nuclear envelope formation, transcription factor processing and prevention of polyglutamine aggregation.^8^, ^9^ The VCP protein has four domains: N‐terminal domain, C‐terminal domain and two ATPase domains, D1 and D2. It has been reported that pathogenic variants in VCP protein induce conformational alterations resulting in significantly perturbed co‐factor interactions and altered ATP binding.^8^, ^9^ In vitro assays have shown that VCP pathogenic mutants have enhanced ATPase activity.^10^, ^11^, ^12^ Strategies to suppress the increased ATPase activity of VCP disease mutants or restoring it to baseline are yet to be developed for clinical trials. Mutations in VCP also affect the consolidation of aggregation‐prone proteins into inclusion bodies and disrupt the autophagic degradation of ubiquitylated proteins, resulting in the accumulation of non‐degradative autophagosomes.^13^, ^14^ Disruption of autophagy due to VCP mutations leads to the buildup of sequestosome‐1 (SQSTM1/p62) and the activated form of microtubule‐associated protein 1A/1B light chain 3B. VCP mutations also lead to the mislocalisation and aggregation of TAR DNA‐binding protein 43 (TDP‐43).^15^, ^16^

Advancements in elucidating the VCP ATPase structure have significantly enhanced our understanding of its regulatory mechanisms, thus helping to identify drug‐like allosteric and ATP‐competitive inhibitors bound to the protein.^17^ Several VCP inhibitors have been reported, including DBeQ, ML240, NMS‐873, NMS‐249, CB‐5083 and CB‐5339. Previous work by our group and others have reported that VCP inhibitors are efficacious in improving VCP‐related MSP1 pathology. Zhang et al.^18^ observed that VCP inhibitors potently rescued disease phenotypes in *Drosophila* and patient fibroblasts. Proteomic analysis by Wang et al.^19^ showed that VCP inhibitors reversed RNA processing and cell cycle‐related dysregulated proteins. Harley et al.^20^ also raised the possibility of leveraging VCP inhibitors that specifically target the D2 ATPase domain as a therapeutic strategy for VCP‐related ALS. Our previous studies using the small molecule allosteric VCP inhibitor CB‐5083 in R155H patient iPSC‐derived myoblasts, and the R155H knock‐in mouse model showed an increase in the expression of autophagic markers and amelioration of muscle weakness, and TDP‐43 expression levels.^21^, ^22^ However, we could not proceed with a patient trial in VCP disease because a phase 1 trial of escalating doses of CB5083 was halted in patients with solid tumours and multiple myeloma due to suspected off‐target activity which led to visual adverse events.^22^ We conducted extensive studies in mice and found that the vision loss is indeed related to inhibition of photoreceptor phosphodiesterase 6, a protein complex family, which is highly concentrated in the retina. The adverse effect of CB‐5083 in the retinae in the mice was seen primarily at high doses of 30 mg/kg and was reversible upon drug discontinuation.^22^

Antisense oligonucleotides (ASOs) have become a promising therapeutic modality for genetic disorders, by directly modulating gene expression at the RNA level.^23^, ^24^ ASOs are short, synthetic nucleic acids, designed to target messenger RNA (mRNA) by Watson‐Crick base pairing, and once bound to the target RNA, can modulate RNA function through multiple mechanisms, including RNase H1‐mediated mRNA degradation and splicing modulation. ASOs have become a top consideration, owing to straightforward validation in disease model systems and demonstrated successes in clinical translation.^25^, ^26^ Leading the way for ASOs currently in medical use is nusinersen which is approved for the treatment of multiple forms of spinal muscular atrophy (SMA).^27^ Currently approved ASO‐based treatments include eplontersen for hereditary transthyretin amyloidosis with polyneuropathy,^28^ olezarsen for familial chylomicronemia syndrome,^29^ eteplirsen for Duchenne muscular dystrophy^30^ and tofersen for superoxide dismutase 1 (SOD1) related ALS.^31^ It was recently reported that an experimental ASO targeting the FUS transcript (ION363) effectively suppressed both wild‐type and mutant FUS expression in the brain and spinal cord of P517L and Δ14 heterozygous mice.^32^ Also, targeted ASO‐mediated Atp1a2 knockdown in astrocytes reduces SOD1 aggregation and accelerates disease onset in mutant SOD1 mice.^33^ Given the gain‐of‐function nature of VCP mutations, we hypothesise that VCP reduction will ameliorate the clinical manifestations of this debilitating disease by normalising VCP activity, thus improving the pathology resulting from the disrupted pathways.

In this study, we evaluate if ASO‐mediated reduction of VCP ameliorates VCP‐related proteinopathy phenotypes in human induced pluripotent stem cells‐derived skeletal muscle progenitor cells (hiPSC‐SMPCs) with the most prevalent *VCP R155H* mutation and in the humanised VCP A232E mice.^2^, ^34^ We found that ASO treatment in VCP *R155H* patient‐derived SMPCs and transgenic mouse models of VCP MSP1 significantly reduced VCP expression, improved autophagy flux and decreased TDP‐43 pathogenesis. These results suggest that knockdown of VCP allele in SMPCs and early in asymptomatic mice could be beneficial in preventing progression of the MSP1 myopathy and holds promise for treatment in patients.

## 2 RESULTS

### 2.1 Successful differentiation of VCP R155H patient‐derived iPSC to myogenic lineage

**FIGURE 1:**
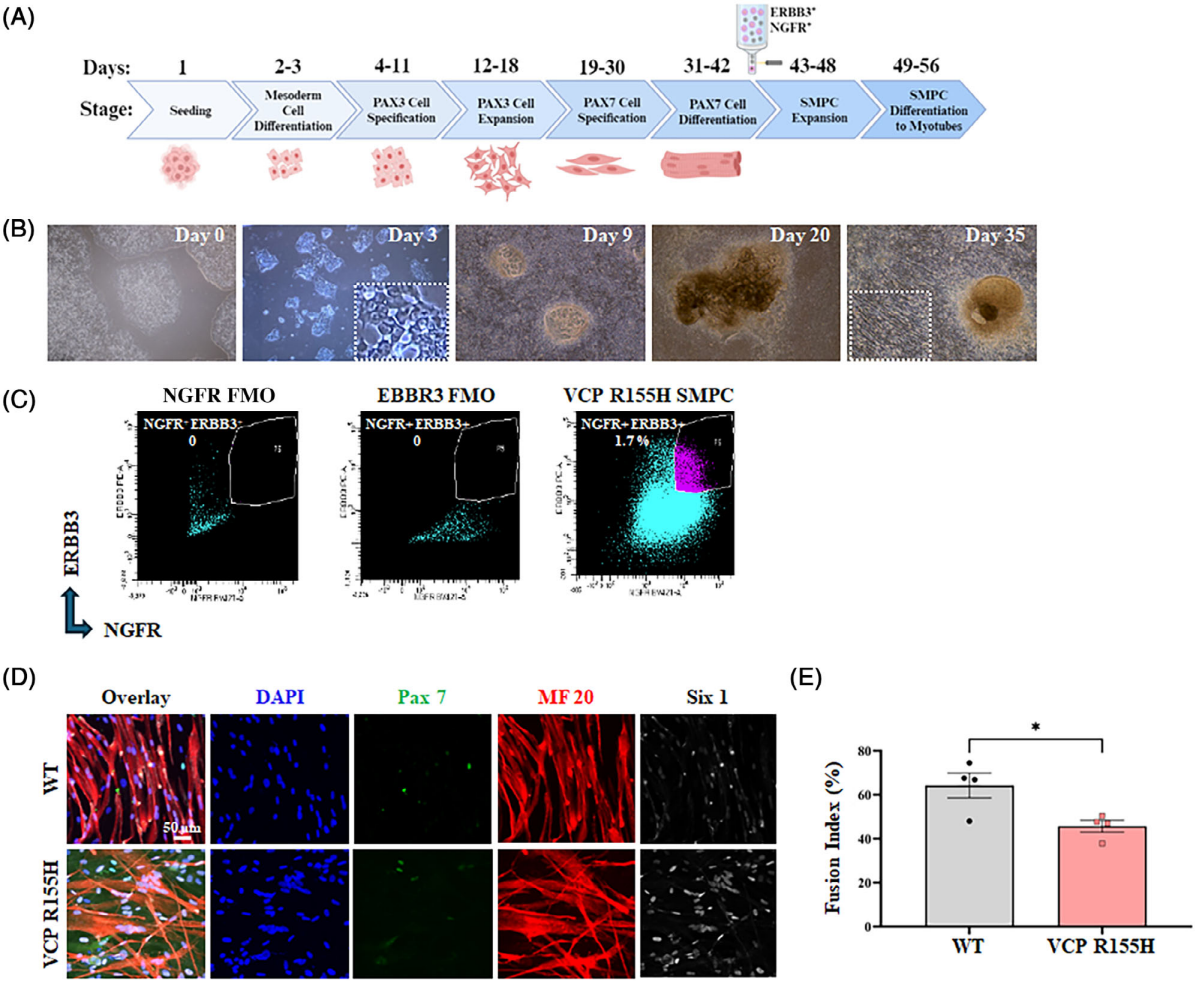
Guided differentiation of patient iPSCs carrying the VCP p.R155H variants to myogenic lineage. (A) Schematic representation for directed differentiation of hiPSCs to skeletal muscle. Created in BioRender. https://BioRender.com/kk798js. (B) Representative brightfield images of the key developmental stages during directed differentiation of hiPSCs to myogenic cells show the formation of distinct 3D structures. Inset shows myotube formation. (C) ERBB3+ and NGFR+ skeletal muscle progenitor cells (SMPCs) were sorted on day 42. FACS plots show percentages of SMPCs sorted from VCP lines. (D) Immunofluorescence microscopy of WT and VCP R155H SMPCs differentiated to myotubes show myosin‐positive multinucleated myofibres along with SIX1 and PAX7 expression. (E) Fusion index percentage of WT and VCP R155H SMPCs differentiated to myotubes. Statistical analysis was performed using multiple unpaired *t*‐test (*p = .05).

We developed a cellular myopathy model of VCP disease by differentiating hiPSC carrying the *VCP p.R155H* variants to SMPCs and assessed the efficacy of VCP ASOs in these cells. Figure 1A illustrates the timeline and major milestones of hiPSC differentiation and maturation into myotubes. It has been previously reported that directed differentiation of any hiPSC line can be achieved through line‐specific tailoring of initial seeding density and addition of the small molecule CHIR99021, an agonist of the Wnt signalling pathway. Indeed, both cell seeding density and Wnt activation play a vital role in mesoderm formation.^35^, ^36^ For VCP hiPSCs, we found that 60 000 cells/cm^2^ and supplementation of 8 µM CHIR99021 for 2 days sufficiently induced mesoderm and later myogenic differentiation, as noted by the formation of distinct 3D structures 1 week after mesoderm induction, and later juxtaposed myotube formation (Figure 1B). At the completion of directed differentiation, enrichment of SMPCs was achieved by selection of the cell population with the highest expression of the cell surface receptors erb‐b2 receptor tyrosine kinase 3 (ERBB3) and nerve growth factor receptor (NGFR) using fluorescence‐activated cell sorting (FACS)^37^ (Figure 1C). We found that co‐expression of ERBB3 and NGFR could sufficiently enrich SMPCs from VCP R155H cells, and these SMPCs robustly expressed Paired box protein 7 (PAX7) (Figures 1D and S1A). SMPCs could be differentiated to form myosin‐positive multinucleated myotubes, suggesting successful enrichment (Figure 1D).^37^ However, VCP R155H SMPCs displayed reduced myotube fusion efficiency compared with the control SMPCs (*p* < .05) (Figure 1E), which may be indicative of their diseased state which we quantified in subsequent figures.

### 2.2 Patient iPSC‐derived SMPCs have elevated levels of TDP‐43 and autophagic markers

**FIGURE 2:**
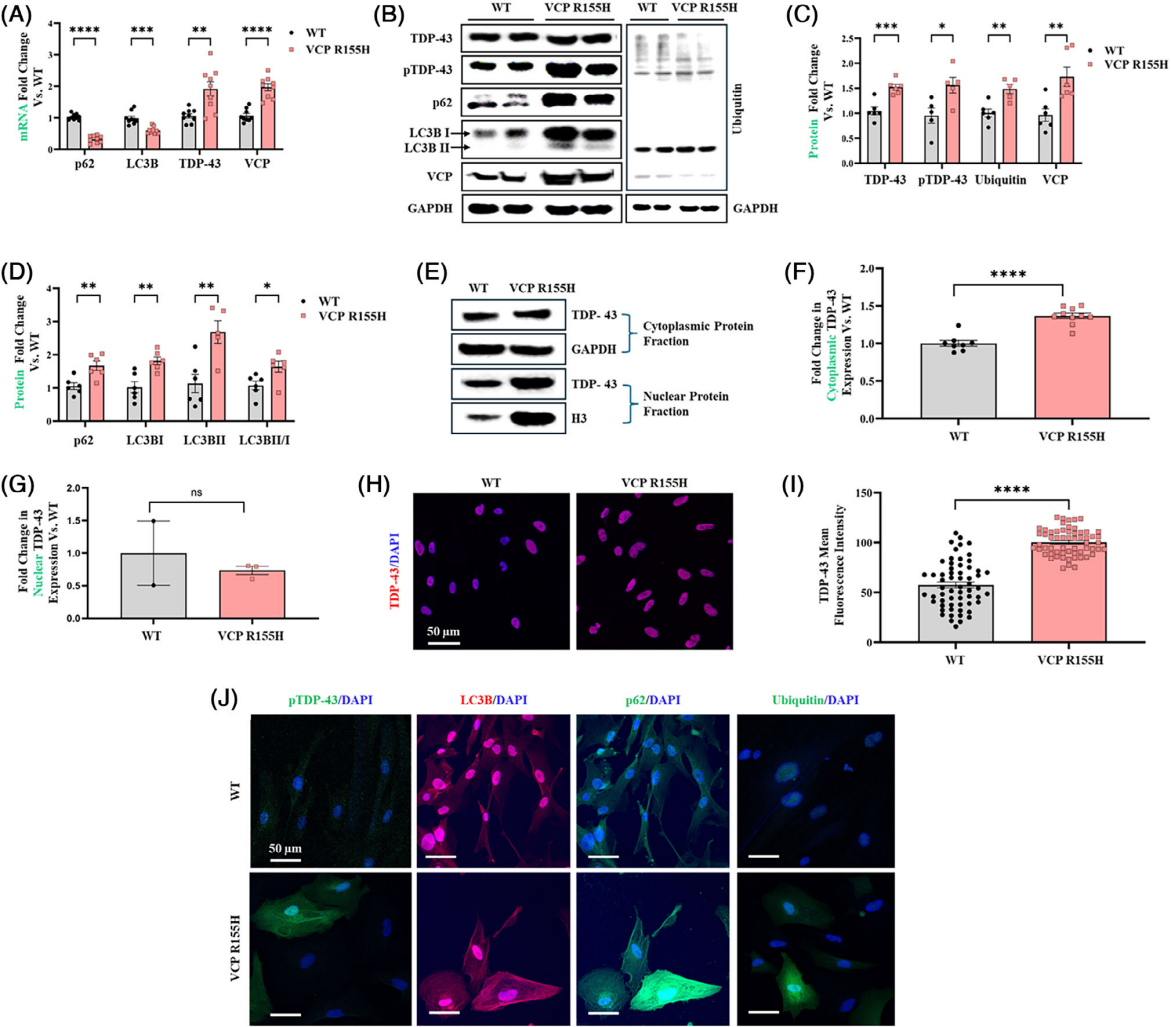
VCP R155H patient iPSC‐derived SMPCs show TDP‐43 pathology, up‐regulated VCP expression and impaired autophagy. (A) Quantitative RT‐qPCR analysis of the VCP R155H iPSC‐derived SMPCs normalised to wild type (WT) and relative to GAPDH. (B) Western blot analysis of protein expression of SMPCs. GAPDH was used as a positive loading control. (C and D) Densitometry analysis of Western blot of VCP R155H patient iPSC‐derived SMPCs normalised to WT and relative to GAPDH. (E) Western blot analysis of cytoplasmic and nuclear fractions of SMPCs. Histone (H3) was used as a positive loading control. (F and G) Densitometry analysis of Western blot of (F) cytoplasmic (*n* = 8–10) and (G) nuclear fraction (*n* = 2–3) of VCP R155H iPSC‐derived SMPCs normalised to WT and relative to H3. (H and J) Immunofluorescence microscopy of WT and VCP R155H SMPCs showed increased TDP‐43, pTDP‐43, SQSTM1/p62, LC3B and ubiquitin positive expression. (I) Mean fluorescence intensity of TDP‐43 expression in VCP R155H iPSC‐derived SMPCs relative to WT. Statistical analysis was performed using multiple unpaired *t*‐tests. *p ≤ .05, **p ≤ .01, ***p = .001 and ****p = .0001 versus WT.

Pathogenic VCP R155H variants are known to lead to the accumulation of ubiquitinated proteins, disruption of autophagy and impaired retrotranslocation of endoplasmic reticulum‐associated degradation substrates.^38^, ^39^ Therefore, we sought to determine the pathophysiological effects of *VCP R155H* pathogenic variants in the patient‐derived SMPCs. It has been reported that dysregulation of protein homeostasis due to variants in VCP may cause TDP‐43 to undergo cleavage, hyperphosphorylation and ubiquitination, leading to its accumulation and aggregation in the cytoplasm.^15^, ^40^, ^41^, ^42^ Phosphorylated TDP‐43 (p‐TDP‐43) is particularly associated with the pathological inclusions seen in TDP‐43 proteinopathies. We found TDP‐43 and VCP gene expression increased significantly by 1.9 ± 0.5‐fold and 1.9 ± 0.3‐fold, respectively, in VCP R155H cells compared with wild type (WT) animals (Figure 2A). The expression of the autophagic proteins sequestome‐1 (SQSTM1/p62) and light chain 3B (LC3B) in VCP R155H cells were 0.3 ± 0.08‐fold and 0.6 ± 0.07‐fold lower, respectively, compared with WT cells, as measured by qRT‐PCR analysis (Figure 2A). In addition, TDP‐43 and p‐TDP‐43 protein levels were significantly increased by 1.5 ± 0.29‐fold and 2.1 ± 1.32‐fold in VCP R155H cells compared with WT cells (Figure 2B, C).

LC3B is a key protein of the autophagy pathway that can be present in two different forms: a cytosolic form (LC3B‐I) and a lipidated form (known as LC3B‐II), where LC3B‐I is conjugated to phosphatidylethanolamine to form LC3B‐phosphatidylethanolamine conjugate, which is bound to the autophagosome membrane.^43^ The ratio of LC3B‐II/I reflect the conversion of LC3B‐I to LC3B‐II upon autophagy activation. We noted increased expression of LC3B‐I by 2 ± 0.73‐fold and LC3B‐II by 3.7 ± 2‐fold, respectively in VCP R155H cells compared with WT (Figure 2B, D). The LC3 II/I ratio clearly showed enhanced autophagic flux in VCP R155H cells. A 1.7 ± 0.5‐fold elevation in the levels of the autophagic marker SQSTM1/p62 also suggested impaired autophagosome‐lysosomal cascade in the VCP R155H cells (Figure 2B, D). Increased VCP protein expression by 1.6 ± 0.39‐fold was also observed in VCP R155H cells (Figure 2B, C). Through nuclear‐cytoplasmic fractionation, we observed significantly elevated cytoplasmic while reduced nuclear TDP‐43 levels by 1.37 ± 0.13‐fold and 1 ± 1.15‐fold, respectively, in VCP R155H SMPCs, implying possible TDP‐43 mislocalisation (Figure 2E–G). In agreement with the Western blot analysis, immunofluorescence data showed significant increase in TDP‐43 (Figure 2H, I) and p‐TDP‐43 (Figure 2J). The VCP R155H cells also showed increased SQSTM1/p62, LC3B and ubiquitin positive expression in the immunofluorescence analysis, therefore consolidating the protein expression data (Figure 2J). The VCP R155H SMPCs thus manifest a phenotype characterised by arrested autophagy and increased TDP‐43 expression, providing a useful model to study the effect of ASO treatment in MSP1 disease.

### 2.3 VCP R155H SMPCs have disrupted lysophagy

**FIGURE 3:**
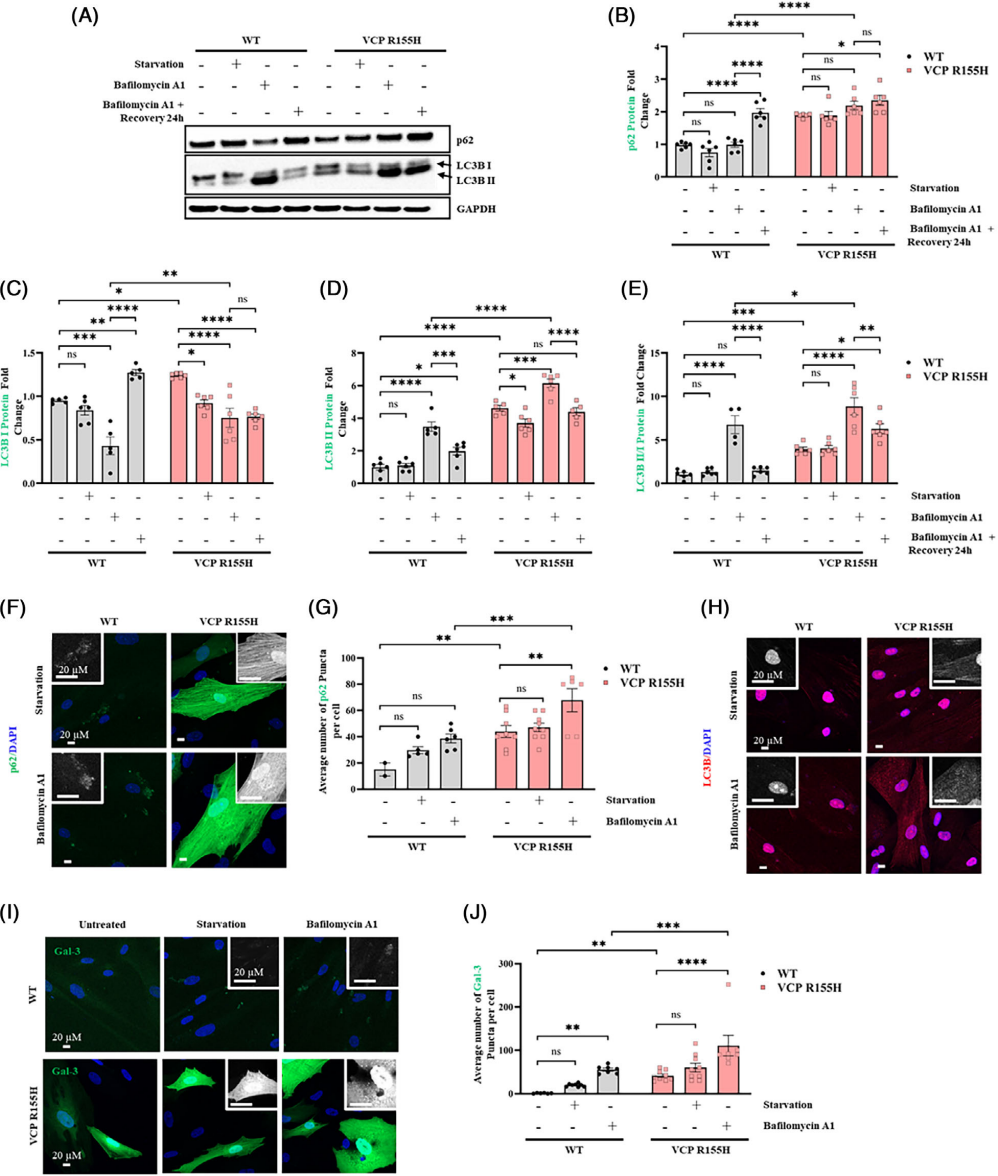
VCP R155H patient iPSC‐derived SMPCs are unable to clear damaged lysosomes. (A) Western blot analysis of WT and R155H iPSC‐derived SMPCs treated with bafilomycin A1 for 3 h, followed by a 24 h recovery period. (B, C, D and E) Densitometry analysis of Western blot analysis of VCP R155H SMPCs treated with bafilomycin A1 normalised to WT SMPCs and relative to GAPDH. (B) SQSTM1/p62, (C) LC3B‐I, (D) LC3B‐II, (E) LC3B‐II/I. (F, G and H) Immunofluorescence microscopy analysis of WT and VCP R155H SMPCs stained with (F) SQSTM1/p62, (H) LC3B and (I) galectin‐3. Inset represents a magnified image of the selected areas. Quantification of average number of puncta per cell in VCP R155H iPSC‐derived SMPCs and WT SMPCs treated with bafilomycin A1 (G) SQSTM1/p62 and (H) galectin‐3. Statistical analysis was performed using two‐way ANOVA followed by Šídák's multiple comparisons test. *p ≤ .05, **p ≤ .01, ***p = .001 and ****p = .0001 versus untreated WT/ VCP R155H); ##*p* ≤ .01, ###*p* = .001 and ####*p* = .0001 versus starvation (WT and VCP R155H); $$$$*p* = .0001 WT versus VCP R155H untreated; &&&&*p* = .0001 bafilomycin A1 treatment corresponding WT versus VCP R155H.

VCP plays a crucial role in maintaining lysosomal homeostasis.^44^, ^45^ The disruption of lysophagy due to VCP pathogenic variants leads to lysosomal accumulation, suggesting a defective lysosomal membrane permeabilisation (LMP) response in the development of VCP‐associated MSP1. VCP R155H and WT cells were subjected to starvation and a combinatorial treatment with bafilomycin A1 (BafA1), an inhibitor of autophagosome‐lysosome fusion, to assess autophagic flux and accumulation of autophagosomes.^46^, ^47^ In VCP R155H cells, impaired autophagy led to a significant increase in SQSTM1/p62 protein levels upon BafA1 treatment (Figure 3A, B). LC3B‐I levels were also significantly higher in BafA1‐treated VCP R155H cells compared with WT (Figure 3A, C). During autophagic induction, LC3B‐II is incorporated into the growing autophagosome membrane, and its level is proportional to the number of autophagosomes in the cell. Western blot analysis revealed that LC3B‐II level in WT did not change upon starvation, but was significantly enhanced after BafA1 treatment, indicating activated autophagic flux (Figure 3A, D). VCP R155H cells also showed a significant increase in LC3B‐II levels upon BafA1 treatment (Figure 3A, D). However, treatment with BafA1 resulted in significantly higher level of LC3‐II and the ratio of LC3B‐II/I in VCP R155H cells compared with WT (Figure 3E). After 24 h of recovery, the LC3B‐II/I ratio returned to basal levels in WT cells (Figure 3E). On the other hand, a higher LC3B‐II/I ratio persisted in VCP R155H cells due to disrupted autophagy (Figure 3E). In line with the Western blot data, immunofluorescence staining showed that the number of SQSTM1/p62‐positive dots was higher in starved VCP R155H cells and significantly increased in the presence of BafA1 (Figure 3F, G). Similarly, starvation led to a marked increase in the number of LC3B‐positive puncta, which was further amplified with BafA1 treatment in VCP R155H cells (Figure 3H).

Lysosomal damage in VCP R155H cells was further examined by analysing galectin‐3 (Gal‐3), a β‐galactoside‐binding lectin that is recruited to the lysosomal membrane solely upon membrane permeabilisation. In WT cells under basal conditions, Gal‐3 exhibited a diffuse cytoplasmic distribution with no detectable puncta (Figure 3I); however, starvation‐induced lysosomal damage resulted in the translocation of Gal‐3, and its consequent binding to compromised lysosomes, forming distinct puncta on the damaged lysosomal membrane. We observed that VCP R155H cells expressed significantly higher Gal‐3 puncta in the basal state (Figure 3I, J). Upon starvation, the number of Gal‐3 puncta increased in both WT and R155H cells due to lysosomal damage. Upon BafA1 treatment the number of Gal‐3 puncta in VCP R155H cells was significantly higher, compared with WT. These results show that VCP R155H patient cells have alterations in lysosomal stability and dynamics.

### 2.4 VCP ASO modulated TDP‐43 pathology and autophagic markers in VCP R155H SMPCs

**FIGURE 4:**
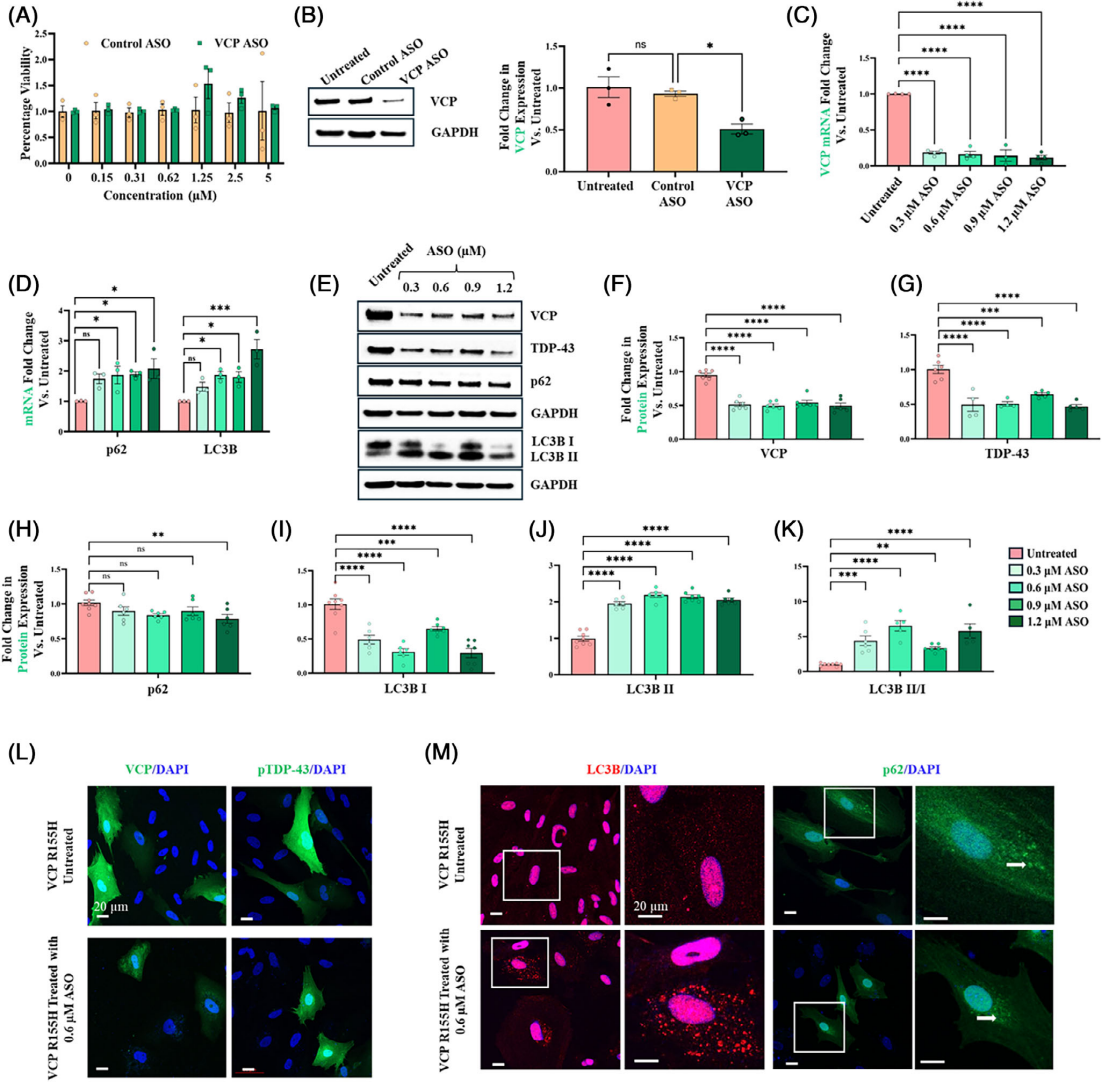
VCP knockdown by VCP ASO and associated effects in VCP R155H SMPCs. (A) Cell cytotoxicity assay by MTT showed good cell viability up to 5 µM VCP ASO. (B) Western blot and corresponding densitometric analysis to quantify VCP protein levels in iPSC‐derived SMPCs treated with 1.2 µM control ASO or VCP ASO. (C–K) Dose–responsive effects of VCP ASO on key molecular hallmarks of MSP1 pathology in patient VCP R155H iPSC‐derived SMPCs following 7 days of treatment. (C) qRT‐PCR for VCP, (D) qRT‐PCR for SQSTM1/p62 and LC3B, (E) Western blot analysis for VCP, TDP‐43, SQSTM1/p62, LC3B‐I, LC3B‐II. (F–K) Densitometry analysis of Western blots in E reports the protein levels of (F) VCP, (G) TDP‐43, (H) SQSTM1/p62, (I) LC3B‐I, (J) LC3B‐II, (K) LC3B‐II/I relative to untreated VCP R155H cells, following normalisation by GAPDH. (L and M) Representative confocal images of immunofluorescence staining in VCP R155H SMPCs, either untreated or treated with 0.6 µM VCP ASO. White arrows indicate the presence of SQSTM1/p62 puncta in SMPCs. Statistical analysis was performed using one‐way ANOVA followed by Dunnett's multiple comparisons test. *p ≤ .05, **p ≤ .01, ***p = .001 and ****p = .0001 versus untreated VCP R155H.

To investigate the therapeutic potential of VCP ASO in rescuing disease phenotypes in VCP R155H cells, we first examined the cytotoxicity of ASOs on the VCP R155H SMPCs at doses ranging from 50 nM to 5 µM. A cell survival rate of ≥98 % was measured in cytotoxicity studies where VCP R155H SMPCs had been treated with ASO up to 5 µM (Figure 4A). No significant differences were observed between control ASO and VCP ASO treatment. The VCP ASO was designed to specifically knock down VCP mRNA using RNase H1‐mediated degradation. Indeed, treatment with 1.2 µM VCP ASO reduced VCP protein expression by 50% (95% CI [19–68]) in the cells, whereas control ASO demonstrated no change in VCP protein levels (Figure 4B). To evaluate potential off target effects, VCP R155H SMPCs were treated with varying concentrations of control ASO. Western blot analysis revealed that control ASO treatment did not affect VCP protein levels (Figure S2A, B). Furthermore, TDP‐43, p62 and LC3B protein expression remained unchanged across all control ASO concentrations, indicating minimal off‐target effects (Figure S2A,C–G). Based on this promising result, we sought to further explore the relationship between different levels of VCP reduction and improvements in disease traits by conducting a dose–response experiment. The SMPCs were treated with four different concentrations of ASOs: 0.3, 0.6, 0.9 and 1.2 µM, to correlate the level of ASO‐mediated VCP mRNA and protein reduction with various molecular signatures that are characteristic of MSP1 disease pathology. We initially treated SMPCs with VCP ASO for 3 days, and Western blot analysis showed no significant changes in VCP, TDP‐43, p62 or LC3B protein levels (Figure S2H, J–N). We hypothesise that the 3‐day treatment period was insufficient to affect these downstream markers, which are characteristic of VCP‐associated pathology. To further investigate, we extended the ASO treatment duration to 7 days. qRT‐PCR analysis and Western blot analysis of 7‐day treatments revealed that ASO treatment resulted in 85% (95% CI [58–93]) reduction in VCP mRNA level and 48% (95% CI [39–56]) reduction in VCP protein expression, respectively, with no significant changes between the different ASO concentrations (Figure 4C, E,F). We observed increased LC3B mRNA and ratio of LC3B‐II/I protein expression upon ASO treatment, which may suggest activation of autophagic flux (Figure 4D, K). Furthermore, VCP ASO treatment resulted in a declining trend (not significant, except for 1.2 µM) of SQSTM1/p62 protein (Figure 4H), despite an increase in its mRNA levels (Figure 4D), which agrees with the notion that SQSTM1/p62 is degraded via autophagy. TDP‐43 protein expression decreased by 60% (95% CI [34–58]) upon ASO treatment compared with untreated cells (Figure 4G). Immunofluorescence confirmed the ASO‐mediated knockdown of VCP expression in VCP R155H cells and associated reduction in pTDP‐43 levels (Figure 4L). Consistent with the Western blot data, the immunofluorescence analysis also showed that VCP ASO treatment resulted in up‐regulation of LC3 and modest down‐regulation of SQSTM1/p62 protein levels, indicating initiation of autophagy (Figure 4M). Total ATPase measurements in VCPR155H SMPCs demonstrated a modest trending toward WT levels following VCP ASO treatment.

### 2.5 VCP ASO restores lysophagy function in VCP R155H SMPCs

**FIGURE 5:**
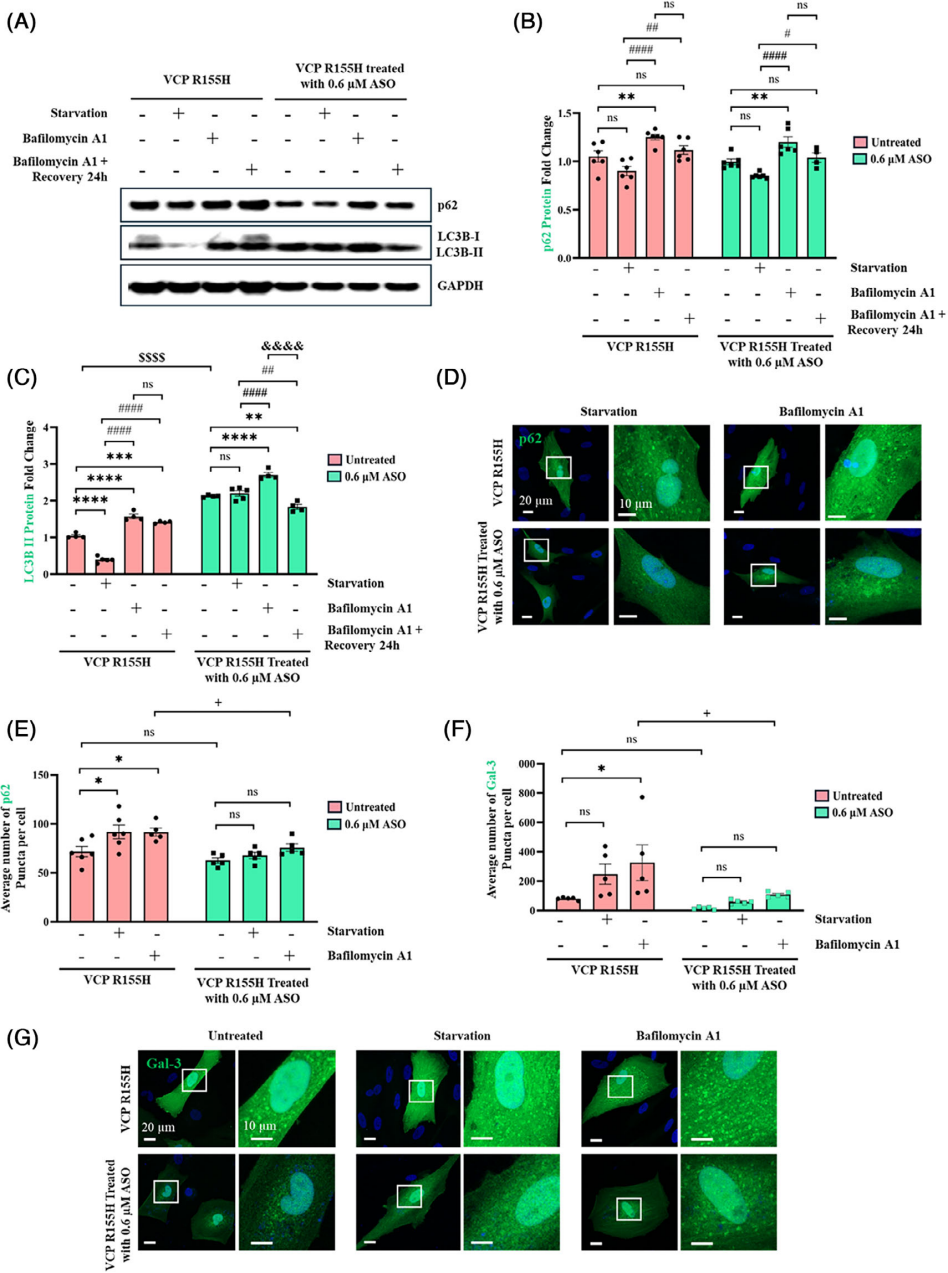
VCP ASO treatment restores lysophagy function in VCP R155H SMPCs. (A) Western blot analysis of R155H iPSC‐derived SMPCs with and without treatment with 0.6 µM VCP ASO for 7 days, followed by 3 h bafilomycin A1 treatment and a 24 h recovery period. (B and C) Densitometry analysis of Western blot analysis of VCP R155H SMPCs treated with 0.6 µM VCP ASO followed by bafilomycin A1 treatment normalised to untreated VCP R155H SMPCs and relative to GAPDH. (B) SQSTM1/p62, (C) LC3B‐II. (D and G) Immunofluorescence microscopy analysis of VCP R155H SMPCs with and without treatment with 0.6 µM VCP ASO followed by bafilomycin A1 treatment, stained with (D) SQSTM1/p62 and (G) galectin‐3. Quantification of average number of puncta per cell in VCP R155H SMPCs with and without treatment with 0.6 µM VCP ASO treated with bafilomycin A1 (E) SQSTM1/p62 and (F) galectin‐3. Statistical analysis was performed using two‐way ANOVA followed by Šídák's multiple comparisons test. *p ≤ .05, **p ≤ .01, ***p = .001, ****p = .0001 versus VCP R155H SMPCs (with and without VCP ASO); #p ≤ .05, ###p = .001 and ####p = .0001 versus starvation in VCP R155H SMPCs (with and without VCP ASO); $$$$p = .0001 VCP R155H with versus without VCP ASO; &&&&p = .0001 bafilomycin A1 treatment in VCP R155H with versus without VCP ASO.

Studies have shown that VCP inhibition in mutant VCP cells rescues defective lysophagy.^48^ We utilised VCP ASO to accelerate clearance and prevent the persistence of damaged lysosomes in VCP R155H cells. We observed a declining trend in SQSTM1/p62 protein expression upon ASO treatment (Figure 4H). To investigate whether p62 degradation was mediated by autophagy, VCP R155H cells were co‐treated with 0.6 µM VCP ASO and Baf A1 (Figure 5A, B). This combination inhibited p62 clearance, which was initially observed with 0.6 µM VCP ASO alone (Figure 4H). Furthermore, p62 expression returned to baseline levels 24 h after Baf A1 washout in VCP R155H cells treated with VCP ASO, implicating autophagic contribution to p62 degradation. Additionally, we noted that the increased expression of LC3B‐II following 0.6 µM VCP ASO treatment was further elevated when autophagosome degradation was blocked by Baf A1 treatment (Figure 5A, C). Immunofluorescence staining also showed BafA1 treatment increased the number of SQSTM/p62 dots in VCP R155H cells treated with 0.6 µM VCP ASO, suggesting blockage in p62 degradation (Figure 5D, E). At basal level, in VCP R155H cells treated with 0.6 µM VCP ASO, Gal‐3 showed uniform cytoplasmic localisation without detectable puncta, signifying the possible removal of damaged lysosomes through autophagy (Figure 5F, G)). VCP R155H cells without ASO treatment had higher Gal‐3 puncta (Figure 5F, G)). Upon starvation and BafA1 treatment, the number of Gal‐3 puncta increased in both treated and untreated VCP R155H cells (Figure 5F, G). VCP being a key regulator of lysophagy, its targeted inhibition by VCP ASO in VCP R155H cells can potentially rescue lysophagic defects.

### 2.6 VCP ASO improves motor function in A232E mice

We conducted in vivo studies using a transgenic humanised mouse model of VCP disease harbouring the most severe mutation, VCP A232E (gifted by Paul Taylor).^49^ To initially generate transgenic mice expressing normal human VCP in all tissues, human VCP cDNA was placed under the control of the CMV‐enhanced chicken beta‐actin promoter permitting widespread VCP expression in muscle, brain and bone. The disease‐associated mutation A232E was then introduced by site‐directed mutagenesis. The resulting transgenic animals were backcrossed onto the C57BL/6 background. The translational value of the VCP A232E transgenic mouse model has been characterised in previous studies.^49^ The mice exhibited progressive muscle weakness; histological analyses of the quadricep muscle revealed signs of myogenic myopathy, such as irregular fibre size, centrally located nuclei and inflammatory cell infiltration; and histopathological analysis of the brain demonstrated ubiquitin‐positive cytoplasmic accumulations of TDP‐43. We additionally performed a natural history study in these mice in our laboratory and found differences between WT and VCP A232E mice in functional studies, including Rotarod, grip strength and inverted screen. Biochemical analysis of quadriceps, tibialis anterior and diaphragm muscle tissue revealed TDP‐43 pathogenesis and impaired autophagy similar to the previous report.^49^

To ensure the absence of ASO‐related toxicity in the mouse studies, 10‐month‐old VCP A232E mice were dosed with either control or VCP ASOs at 50 mg/kg, once a week for 12 weeks, via subcutaneous injection. Age and sex‐matched WT mice were used as a reference control (Figure S3A, B). Percent change of body weights, illustrating relative changes in body weight over time, was calculated from a 6‐month‐old baseline (pre‐treatment) and set to zero for visualisation. At the end of the 12‐week dosing regimen, liver, spleen and kidneys were collected from the mice, and their weights were measured and analysed for potential toxicity. The serum levels of the liver transaminases aspartate aminotransferase (AST) and alanine aminotransferase (ALT), creatine kinase (CK), as well as the levels of blood urea nitrogen (BUN) whose elevation (more than twofold) would indicate concerning liver, muscle or renal toxicity, were also measured (Figure S3B–H). We observed no significant changes in organ weights or serum parameters between VCP ASO and control ASO treatment groups at 12 weeks following injections using Dunnett's multiple comparisons test, except for BUN levels; however, all levels remained in the normal range (Figure S3B–H). Therefore, we conclude that under the experimental conditions used, the VCP ASO was well tolerated in mice.

**FIGURE 6:**
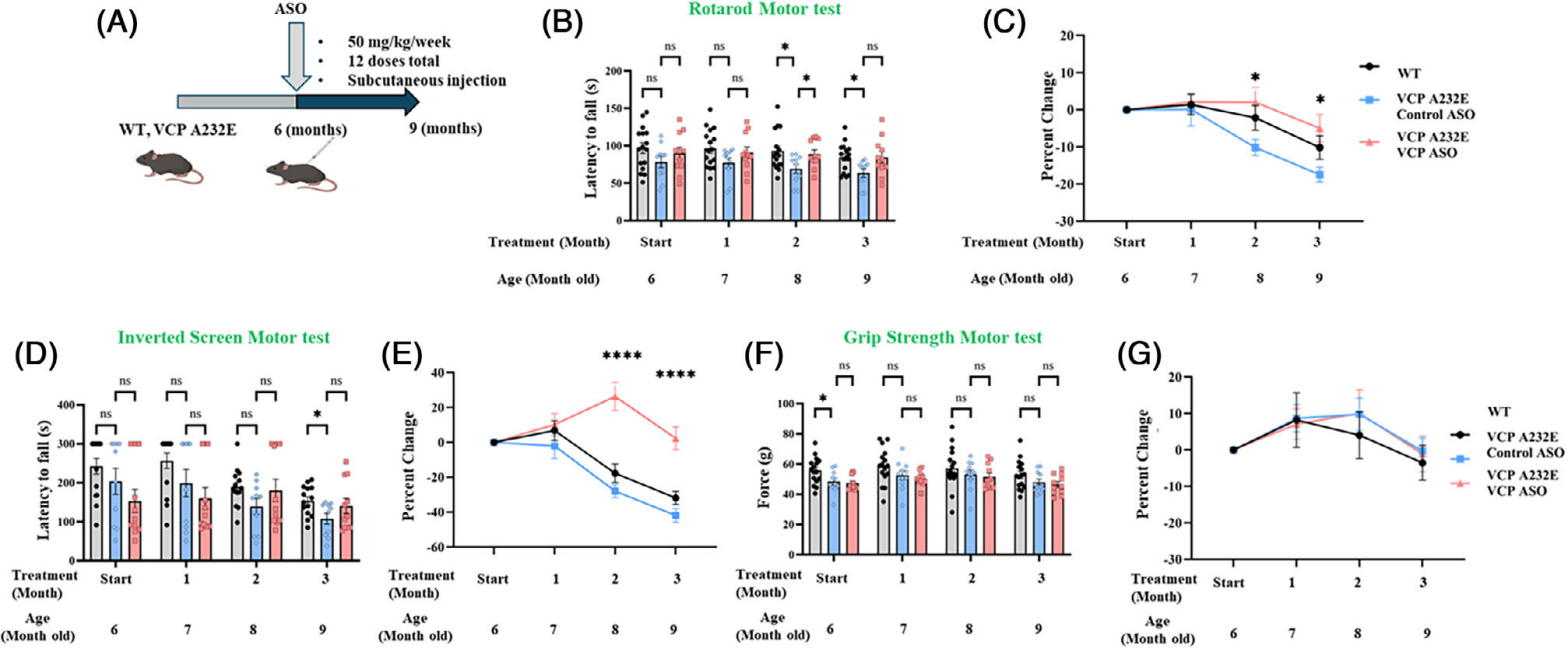
Motor testing analysis of WT and VCP A232E mice treated with VCP ASO and control ASO. (A) Schematic illustration of experimental design. 6‐month‐old VCP A232E mice (n = 11–16) were dosed weekly by subcutaneous injection with control ASO or VCP ASO (50 mg/kg/week) for a total of 3 months. (B, D and F) Rotarod, inverted screen testing and grip strength, in WT mice and A232E mice treated with either control ASO or VCP ASO. Testing started at 6 months of age and was performed monthly for 3 months. (C, E and G) Raw data were analysed in addition to percent change from baseline testing in the different groups and normalised with control ASO group for comparison. (C) Percent change in Rotarod, (E) percent change in inverted screen and (F) percent change in grip strength. The 6‐month‐old data point represents baseline motor performance prior to ASO treatment and set to zero for visualisation. Statistical analysis was performed using two‐way ANOVA followed by Dunnett's multiple comparisons test. *p ≤ .05 and ****p ≤ .0001 versus control ASO ns: not significant.

Motor strength and coordination were assessed monthly in VCP A232E mice that received subcutaneous injections of 50 mg/kg VCP ASO or control ASO once a week from 6 to 9 months of age (Figure 6A). The 6‐month timepoint represents baseline motor performance prior to ASO treatment. A group of age‐matched untreated WT mice was included in the study as reference controls. Motor performance in the different groups of mice was evaluated with the Rotarod, grip strength and inverted screen assays, and both raw data and percent change from the respective baseline values were analysed. VCP A232E mutant mice dosed with VCP ASO showed significant improvement in Rotarod performance compared with control ASO‐treated A232E mice at 2 months of treatment (Figure 6B). Motor testing data trended for improved performance in the inverted screen test with VCP ASO treatment in A232E mice but did not reach statistical significance (Figure 6D). Percent change analysis of rotarod and inverted screen tests showed significant improvement in VCP ASO‐treated mice after 2 and 3 months of treatment when compared with the control ASO‐treated mice (Figure 6C, E). While the absolute differences between time points are modest (Figure 6B, D), the percent change graph highlights relative trends in motor performance over time. There was no change in grip strength performance (Figure 6F, G). To provide an integrated view of the cumulative effect of ASO treatment across the intervention period, we conducted statistical analysis using area under the curve (AUC) tests for the rotarod, inverted screen, grip strength and their respective percent change analyses. We compared the three groups AUCs using one‐way ANOVA and found a significant difference in inverted screen percent change AUC between VCP A232E treated with VCP ASO and control ASO (*p* < .0001). We did not find significant differences of AUCs between VCP ASO and control ASO‐treated groups for rotarod, inverted screen, grip strength, rotarod percent change or grip strength percent change (Figure S4A–F).

### 2.7 VCP ASO ameliorates myopathy and biochemical abnormalities in the A232E mouse model of MSP1 disease

**FIGURE 7:**
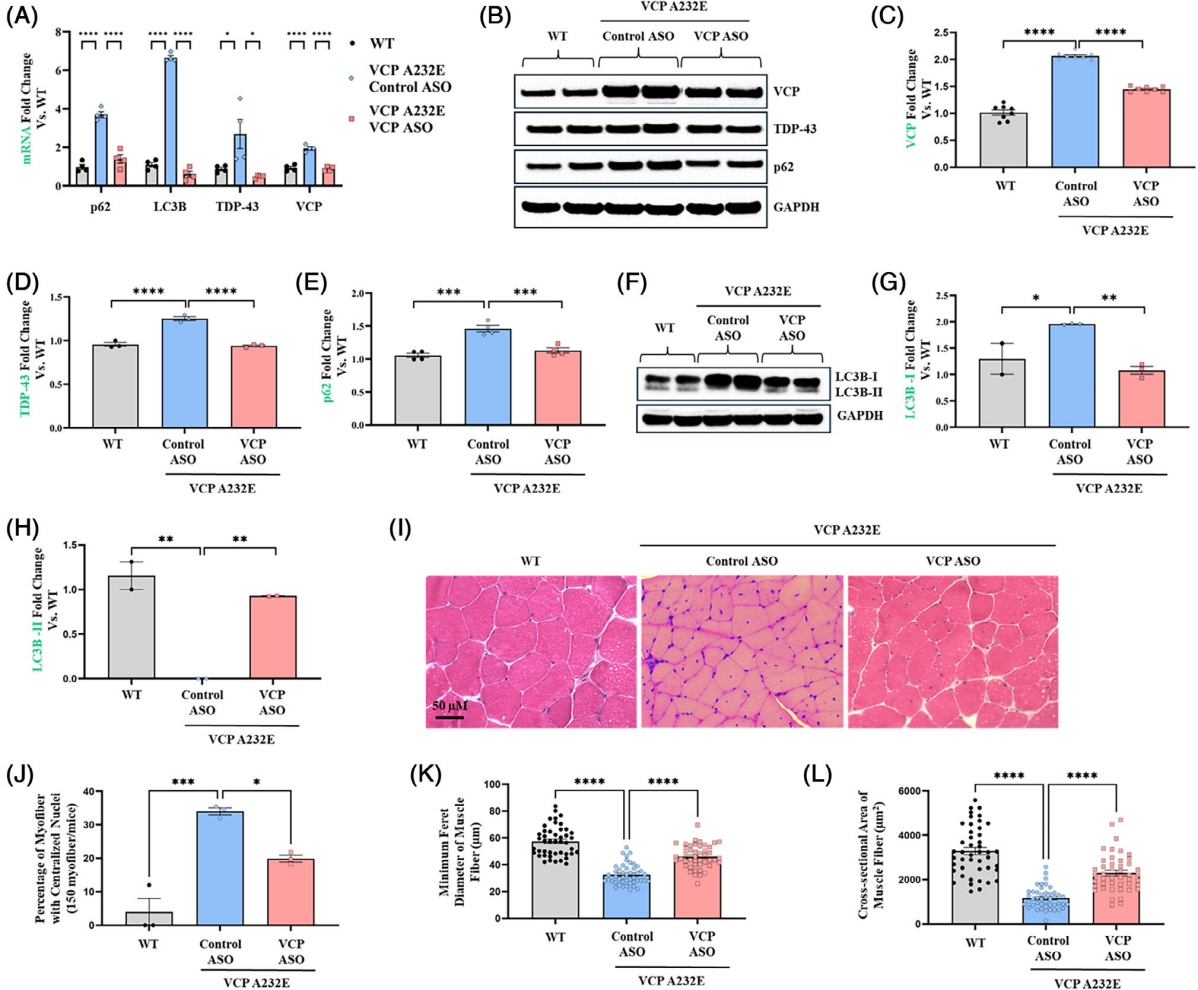
Improvement of disease hallmarks in VCP A232E mice dosed with VCP ASO. (A) VCP ASO treatment reduced VCP mRNA levels in quadriceps. (B and F) Western blot analysis of VCP and other proteins in the autophagy pathway. (C, D, E, G and H) Densitometric analysis of Western blot shown in panel (B). (C) VCP, (D) TDP‐43, (E) p62, (G) LC3B‐I, (H) LC3B‐II and (I) H&E staining of myofibril structure in histological sections of quadriceps muscle collected from WT or A232E mice dosed with control ASO or VCP ASO at the end of the 3‐month study. (J) Quantification of the centralised nuclei (150 myofibres from 3 mice per group). (K) Minimum Feret diameter of muscle fibre and (L) cross‐sectional area of muscle fibre in the histological sections of quadricep muscle from VCP A232E mice dosed with 50 mg/kg ASO for 3 months. One‐way ANOVA test followed by Dunnett's multiple comparisons test was used for qPCR and WB densitometry quantification. Unpaired *t*‐test was used for central nuclei quantification (*n* = 3 mice per group). *p = .05, **p = .01, ***p = .001 and ****p = .0001 versus control ASO (*n* = 3 mice per group).

Taylor et al.^49^ reported that VCP A232E mice muscle exhibit pathological signs of myogenic myopathy, such as fibre size irregularity, centralised nuclei, inflammation and TDP‐43‐positive intranuclear and cytoplasmic aggregates. Consistent with those reports, we observed that 9‐month‐old VCP A232E mice treated with control ASO had significantly higher TDP‐43 mRNA (2.69 ± 1.4‐fold elevation) and protein (1.3 ± 0.07‐fold increase) expression in quadriceps muscle compared with WT littermates. Treatment with VCP ASO significantly reduced TDP‐43 mRNA levels by 0.47 ± 0.08‐fold and protein expression by 0.75 ± 0.03‐fold compared with control ASO in quadriceps muscle of VCP A232E mice (Figure 7A, B, D). SQSTM1/p62 and LC3B mRNA expression were 4 ± 0.98‐fold and 6.6 ± 0.2‐fold higher, respectively in quadriceps muscle of VCP A232E mice treated with control ASO compared with WT (Figure 7A). Treatment with VCP ASOs decreased SQSTM1/p62 and LC3B mRNA expression by 1.3 ± 0.43‐fold and 0.6 ± 0.3‐fold, respectively compared with control ASO treatment (Figure 7A). Similarly, SQSTM1/p62 and LC3B protein levels were reduced by 22% (95% CI [13–31]) and 45% (95% CI [32–55]) following VCP ASO dosing in quadriceps muscle of VCP A232E mice compared with control ASO (Figure 7B, E–H). The conversion of the soluble LC3B‐I protein to its lipidated, membrane‐bound LC3B‐II form represents a critical step in autophagosome formation. Therefore, the absence of LC3B‐II in control ASO‐treated VCP‐A232E mice indicates an impairment in the early stages of autophagy (Figure 7H). Additionally, VCP ASO dosed for 12 weeks at 50 mg/kg via subcutaneous injection resulted in 0.88 ± 0.1‐fold knockdown of VCP mRNA and a 30% (95% CI [27–32]) reduction in VCP protein expression compared with control ASO in quadriceps muscle of VCP A232E mice (Figure 7A–C). Haematoxylin and eosin (H&E) staining of histological sections of quadriceps muscle from VCP A232E mice dosed with VCP ASO showed improved myofibre morphology compared with control ASO (Figure 7I). Indeed, WT mice had homogeneously sized myofibres (Feret's diameter and cross‐sectional area: 57 ± 3.28 µm and 3289 ± 323 µm^2^) with peripherally located nuclei (Figure 7J,–L), whereas 9‐month‐old VCP A232E mice dosed with control ASO showed cellular infiltrations into the interstitium of the myofibres along with a significantly increased number of centralised nuclei, consistent with MSP1 myopathy (Feret's diameter and cross‐sectional area: 32 ± 2.23 µm and 1163 ± 143 µm^2^) (Figure 7K, L). VCP ASO treatment significantly decreased the number of central nuclei compared with control ASO (WT: 12 ± 2% myofibres had centralised nuclei, control ASO: 34 ± 2% myofibres had centralised nuclei, VCP ASO: 20 ± 2% myofibres had centralised nuclei; over 150 myofibres examined from 3 mice per group) (Figure 7J). VCP ASO treatment also significantly increased Feret's diameter and cross‐sectional area by 46 ± 2.56 µm and 2295 ± 250 µm^2^ respectively, indicating potential therapeutic benefit.

## 3 DISCUSSION

VCP autosomal dominant multisystem proteinopathy caused by gain‐of‐function missense variants is associated with different levels of hyperactive enzymatic activity, which in turn are associated with clinical variability in the phenotype.^10^, ^11^, ^12^ To ameliorate the gain‐of‐toxicity, an ideal approach is to reduce the expression of VCP mutant proteins. While previous reports have indicated that small molecules improve the phenotype in preclinical studies in drosophila, patient derived myoblast cells and in the VCP R155H mouse model,^18^, ^21^, ^22^ the compounds used were not approved for patient trials requiring a long duration of treatment for this chronic disease. A phase I trial for cancer was terminated because of adverse effects on vision, such as photophobia and dyschromatopsia.^22^ To address this shortcoming, we identified a potent ASO targeting VCP RNA that safely knocks down human VCP expression by virtue of Rnase H1‐mediated mRNA degradation. ASO treatment in functionally validated iPSC‐derived VCP R155H SMPCs and transgenic mouse models of VCP disease significantly reduced VCP expression, improved autophagy markers and decreased TDP‐43 pathogenesis. Our study provides proof of principle that reducing the expression of VCP using ASOs mitigates the toxic effect of the protein, restores homeostasis and ameliorates VCP‐associated disease pathology. Consistent with prior findings by Custer et al.^49^ showing cytoplasmic accumulation of TDP‐43 in skeletal muscle of 9‐month‐old VCP mutant mice, our laboratory has similarly observed mislocalisation of TDP‐43 from nucleus to cytoplasm in quadriceps of 10‐month‐old VCP‐A232E mice. These observations reinforce the pathological similarity between this mouse model and human VCP disease. Based on our findings, we propose that VCP‐targeting ASOs could be a potential therapeutic approach for reducing pathogenic VCP expression in patients.

ASOs are uniquely tailored to specifically correct the root cause of the disease at the RNA level, offering a strategic alternative therapeutic strategy over treatments focused on downstream processes.^24^ Many antisense drug candidates are presently in clinical trials for a broad spectrum of diseases, including endocrine, metabolic, cardiovascular, neurological, inflammatory, neuromuscular and infectious disorders.^24^ Currently, no antisense therapeutics have been approved by the United States Food and Drug Administration (US FDA) for the treatment of VCP disease‐associated MSP1. Additionally, as the VCP function is indispensable for cellular functions, therapeutic strategies for MSP1 should ideally focus on selective modulation of VCP activity, balancing efficacy with safety.

We assessed the effects of ASOs, specifically targeting the human VCP gene in the patient iPSC‐derived SMPCs bearing the most common VCP R155H/+ disease variant and the humanised VCP A232E/+ mouse model. The VCP R155H and A232E mutation, located in the N domain and D1 ATPase domain, respectively, destabilise the interaction between the N and D1 domains through steric hindrance, interfering with VCP's ability to interact with substrates and cofactors, thereby compromising protein quality control mechanisms.^10^ In our laboratory, patient iPSC‐derived SMPCs were characterised and were shown to have higher protein expression levels of the autophagy markers LC3‐II/I, SQSTM1/p62, VCP and TDP‐43, as previously reported.^21^, ^50^ These cellular and molecular alterations make them a suitable model to assess the therapeutic potential of ASOs. We observed discrepancy between reduced SQSTM1/p62 and LC3B mRNA levels but increased protein expression in VCP R155H cells likely reflecting impaired autophagic flux. Defective autophagosome–lysosome fusion leads to reduced degradation and accumulation of LC3B‐II and p62 proteins despite lower transcript levels. Additionally, feedback inhibition from LC3B‐II buildup may suppress LC3B transcription, consistent with disrupted autophagy in VCP‐mutant cells.

The response to lysosomal damage is emerging as a critical cellular mechanism for maintaining homeostasis, and its dysfunction may play a role in VCP disease pathogenesis.^51^, ^52^ Efficient lysosome turnover relies on a coordinated sequence of events, including the VCP‐dependent removal of ubiquitylated substrates.^53^ The absence of functional VCP may disrupt the clearance of ubiquitinated substrates from compromised lysosomes and initiation of autophagy. We found that BafA treatment, which disrupts the lysosomal degradation pathway, causes accumulation of SQSTM1/p62 and increased LC3B‐I to LC3B‐II conversion in R155H VCP cells compared with WT. We further observed that VCP mutation disrupts the clearance of Gal‐3‐positive damaged lysosomes in VCP R155H cells. Other studies have also reported R155H VCP mutations lead to acute LMP and impair the cell's ability to repair damaged lysosomes.^48^, ^54^

We showed that ASO treatment was effective in targeting VCP mRNAs and reduced VCP protein expression by 0.6‐fold in SMPCs compared with untreated SMPCs. Zhang et al.^55^ reported that the R155H mutation causes ∼twofold increase in VCP's ATPase activity. A decrease in VCP expression upon ASO treatment would consequentially reduce VCP's ATPase activity; however total ATPase measurements in VCPR155H SMPCs demonstrated a modest trending toward WT levels following VCP ASO treatment, supporting the expected functional impact of partial VCP knockdown. In human motor neurons, enhanced D2 ATPase activity caused by mutant VCP has been implicated as a contributing factor to the mislocalisation of TDP‐43.^20^ ML240 and CB‐5083 selective VCP inhibitor reversed the mislocalisation of TDP‐43 by pharmacologically inhibiting the D2 ATPase domain of VCP protein.^20^ In agreement, we also found VCP ASO decreased TDP‐43 expression in VCP R155H cells. Future studies utilising standardised imaging and quantitative analysis methods will be valuable for more precise assessment of TDP‐43 localisation. Corroborating earlier findings, we observed a steady up‐regulation of autophagy‐related markers following VCP knockdown.^56^ Lee et al.^57^ also suggested that silencing VCP via short interfering RNA (siRNA) increases the LC3B‐II/I ratio, and this increase was further enhanced following BafA1‐mediated inhibition of autophagosome degradation. However, we observed an atypical response at 0.9 µM of VCP ASO which may represent a concentration‐dependent mechanistic effect. This non‐monotonic behaviour could arise from partial RNase H saturation, compensatory transcriptional feedback or limitations in ASO uptake and processing at intermediate doses. Additionally, dose‐dependent splicing or off‐target interactions may contribute to the observed variability.

We observed no significant adverse effects from VCP ASO administration in mice. Additionally, organ analysis confirmed no signs of local or peripheral toxicity in the liver, spleen and kidney. Due to limited serum available, we focused on key liver and renal safety markers and did not identify concerning toxicity. Muscle pathology assessments showed that the characteristic abnormalities in VCP A232E mice were substantially reduced following VCP ASO treatment. Targeting of VCP mRNA by the ASO resulted in widespread down‐regulation of VCP protein expression in the quadriceps muscle of VCP A232E mice.

ASOs have emerged as a promising RNA‐targeting strategy for neurological diseases, represented by the recent US FDA approval of tofersen, an ASO designed to reduce SOD1 mRNA in ALS patients carrying SOD1 mutations. Tofersen demonstrated improvements in disease biomarkers by lowering production of the toxic SOD1 protein.^58^ In our results, ASO treatment led to a reduction in TDP‐43 pathology, which was accompanied by improved behavioural outcomes in VCP A232E mice, highlighting the therapeutic potential of reducing TDP‐43 dysregulation in multisystem proteinopathy. This is consistent with prior studies in ALS/FTD models showing that ASO‐mediated suppression of TDP‐43 improves behavioural dysfunction, indicating their therapeutic potential for TDP‐43‐related disorders.^59^ In contrast to our in vitro data, in vivo treatment of VCP ASO led to reduction in autophagy markers SQSTM1/p62 and LC3B. We hypothesise that the 3‐month ASO regimen mitigated protein aggregation, leading to a decreased necessity for autophagy activation in vivo. The molecular changes induced by the ASO treatment thus led to improvement of motor function in A232E mice. Only male mice were used in the in vivo ASO treatment studies to reduce variability, because the VCP A232E transgene is X‐linked and X‐chromosome inactivation in females could introduce greater variability. In future studies, treatment of both sexes is planned to evaluate potential sex‐specific differences in treatment response.

Studies have shown that blocking ATP hydrolysis not only counteracts the downstream effects of excessive hydrolysis but also intensifies dominant‐negative impacts on ATPase function.^20^ Restoring VCP ATPase activity toward wild‐type levels represents a key translational goal, given the about twofold hyperactivity conferred by the R155H mutation.^55^ Several complementary strategies could be pursued in follow‐up studies. First, allele‐specific ASOs could preferentially silence the mutant transcript, preserving wild‐type expression as demonstrated in other dominant neurodegenerative disorders. Additionally, combinatorial approaches with small‐molecule VCP inhibitors offer a rational means of fine‐tuning activity. In particular, our prior work with CB‐5083 demonstrated that long‐term dosing in VCP R155H homozygous mice was well tolerated and reduced pathological biomarkers including TDP‐43 and p62, while improving muscle pathology without ocular toxicity.^21^ Such combination strategies could enable more precise modulation of ATPase activity while minimising the risk of over‐suppression. Considering the wide functional scope of VCP, it is plausible that VCP mutations not only exert dominant gain‐effects but may also exert loss of function effects, influenced by specific cofactor associations and downstream signalling cascades.

An additional consideration for the translational development of VCP‐targeting ASOs is biodistribution in view of the brain and spinal cord involvement in this disease. ASOs are large, negatively charged molecules that do not efficiently cross the blood–brain barrier. Since MSP1 manifests with variable involvement of skeletal muscle, bone and the central nervous system, therapeutic delivery will likely need to be tailored by phenotype. Systemic administration is optimal for skeletal muscle and peripheral pathology, as modelled in the present study, whereas CNS involvement may require intrathecal delivery approaches, similar to those successfully applied in other ASO therapies such as nusinersen for SMA.^60^, ^61^ These strategies provide a clear framework for addressing both peripheral and CNS disease compartments in future work.

**FIGURE 8:**
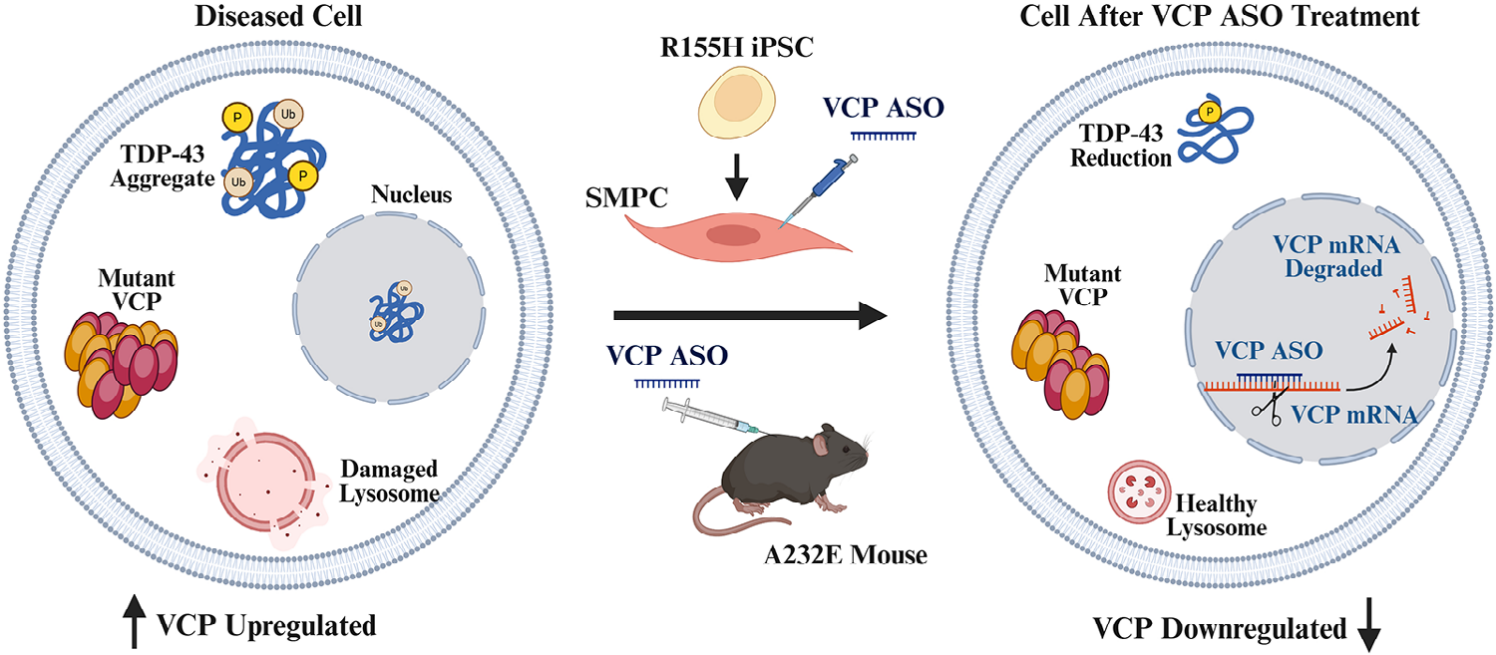
Model of pharmacological intervention with VCP ASO in VCP R155H patient‐derived SMPCs and A232E transgenic mouse model of VCP MSP1. The proposed therapeutic approach involves an ASO specifically designed to target VCP mRNA through Watson‐Crick base pairing. Upon binding, the ASO modulated RNA function via mechanisms such as RNase H1‐mediated mRNA degradation and splicing modulation. Reduced VCP protein expression attenuated TDP‐43 pathology and restored autophagy flux, leading to recovery of healthy lysosomal function.

In summary, our study indicates that VCP‐targeting ASOs are a safe and potentially efficacious treatment for reducing VCP expression, improving autophagy flux, decreasing TDP‐43 pathogenesis and mitigating muscle pathology in VCP‐associated disease (Figure 8). The advancement of VCP inhibitors into phase I clinical trials for cancer acknowledge their potential for other debilitating and presently incurable disorders.

## 4 MATERIALS AND METHODS

### 4.1 Differentiation procedure of iPSCs to SMPCs

We differentiated iPSCs of one clinically affected female patient (aged 65 years) carrying the VCP p.R155H variants to SMPCs using a previously published protocol.^35^ The patient developed myopathy with onset at the age of 33 years; Paget disease with onset at 37 years of age. Briefly, iPSCs were cultured on mTESR^+^ for at least three passages. After pre‐treating the iPSCs for 1 h with 10 µM Rho associated kinase inhibitor (rock inhibitor; RI) (Stem cell Technologies) supplemented media, cells were dissociated, seeded at a concentration of 550 000 cells/well (based on our standardisation protocol) on Matrigel‐coated plates and grown in mTeSR^+^ with RI media. On days 2 and 3, cells were treated with 8 µM CHIR (Tocris Biosciences) supplemented in E6 medium. Afterwards, the culture medium was changed, and cells were cultured in E6 medium until day 11. Cells were then switched to StemPro‐34^+^basic fibroblast growth factor media for an additional 7 days. The culture medium was then changed to E6 medium supplemented with IGF1 for 7 days. The cells were then fed Dulbecco’s modified Eagle medium (DMEM/F12) with N‐2 Supplement (Gibco), insulin–transferrin–selenium (ITS; Gibco) and IGF1 media for 5–7 days and thereafter SB431542 (Sigma) was added for an additional 7 days. FACS was then conducted using mouse anti‐human fluorochrome‐conjugated monoclonal antibodies of erbB3/HER‐3 (Biolegend; 324705) and NGFR (CD 271; BD Pharmingen™ 562123). Briefly, the cells were dissociated as single cells, incubated in human Fc block antibody to block non‐specific binding of Fc, and subsequently fluorochrome‐conjugated antibodies were added according to previously published protocol.^35^ After 45 min incubation, the cell–antibody conjugates were washed with FACS buffer (2% fetal bovine serum in sterile phosphate‐buffered saline [PBS]) and analysed with BD ARIA II (BD Biosciences) flow cytometer and analysed with the BD FACS Diva 8.0.2 software. Matched fluorophare‐conjugated isotype control antibodies were used to set control gates. The sorted SMPCs were expanded in SMPC expansion media SkGM‐2 Bullet Kit (Lonza) for characterisation and further experimentation. The SMPCs were differentiated to myotubes by seeding them on Matrigel‐coated plates and culturing them in DMEM/F12 medium supplemented with N‐2 Supplement, ITS, IGF1 and SB431542 for 5–7 days. To evaluate the differentiation and myotube formation ability of SMPC, cultures were immunostained with MF 20 and PAX7 antibodies. The number of nuclei contained within each myotube was counted and the fusion index was calculated as the percentage of nuclei in myotubes over total nuclei.^37^ At least three random images were taken per well, and nuclei were counted using ImageJ.

### 4.2 Identification of ASOs targeting human VCP

The ASOs used in the studies described here were 16 nucleotides in length with a phosphorothioate backbone and three 2′‐constrained ethyl (cEt)‐modified nucleotides at both ends (3–10–3 gapmer configuration). The ASOs were either unconjugated or conjugated to a lipid (C16, palmitate) at the 5′ end via a phosphodiester linkage. For in vitro studies we had originally used conjugated ASOs, since those compounds became available first. The C16‐conjugated ASOs resulted in good activity in cells without toxicity. Unconjugated ASOs were used for in vivo studies where they successfully resulted in good activity without the need for lipid conjugation, so we decided to use the unconjugated ASOs in the in vivo setting to evaluate systemic delivery and biodistribution without unnecessary modifications. A well‐characterised control ASO which does not hybridise to any mouse mRNA sequence was included in the experiments. The oligonucleotides were synthesised and purified as previously described.^62^ Of the 150 ASOs targeting VCP that were initially screened for knockdown efficiency and toxicity, 10 were further evaluated in vivo for tolerability. These ASOs were administered subcutaneously to 7‐week‐old wild‐type CD‐1 male mice at a dose of 50 mg/kg weekly for 4 weeks, followed by sacrifice 72 h after the final dose (data not shown). The best performing ASOs that demonstrated the most favourable balance between efficacy and safety were chosen for further experiments. Specifically, selection was based on knockdown efficiency of VCP protein and serum biomarkers of organ toxicity.

### 4.3 MTT assay

Cell cytotoxicity upon ASO treatment was measured using the 3‐(4,5‐dimethylthiazol‐2‐yl)‐2,5‐diphenyl‐2H‐tetrazolium bromide (MTT) assay (Sigma). Briefly, 1 × 10^4^ SMPCs per well were seeded in a 96‐well plate and treated with control ASO or VCP ASO at concentrations ranging from 50 nM to 5 µM for 48 h. The treatment was conducted in serum‐reduced optiMEM medium overnight. The culture medium was then replaced with complete SMPC medium the following day. Cells were then incubated with 5 mg/mL MTT for 3 h at 37°C. A total of 100 µL of DMSO was added to each well and the culture plate placed on a shaker for 30 min to dissolve the formazan crystals, resulting in the formation of a coloured solution. The amount of formazan produced in each well was quantified by measuring the absorbance of the coloured solution at a wavelength of 570 nm. Three independent replicate measurements were collected and reported as the mean value ± standard deviation.

### 4.4 ASO treatment of SMPCs

SMPCs were transfected with 300, 600, 900 and 1200 nM ASOs using lipofectamine 3000 (Thermofisher Scientific). Transfection was done in serum‐reduced OptiMEM medium overnight and replenished with complete SMPC medium the following day. Cells were treated with ASOs for either 3 or 7 days. For the 3‐day treatment, transfection was performed on day 1, and cells were collected on day 3. For the 7‐day treatment, transfections were conducted on days 1, 3 and 5. In parallel, cells were treated with control ASO for 7 days following the same transfection regimen. After 7 days of treatment, the cells were either fixed for immunocytochemistry or lysed for total RNA and protein extraction. To test autophagy flux, cells were incubated in serum‐free medium for 24 h to induce starvation, then treated with 10 nM BafA1 (Sigma–Aldrich; B1797) for 3 h prior to harvesting, following previously published protocol.^63^

### 4.5 RT‐qPCR

Total RNA was extracted from SMPCs, ASO‐treated SMPCs and mouse tissues (quadriceps) using the Monarch Total RNA kit (New England Biolabs), and the High‐Capacity cDNA Reverse Transcription kit (Thermo Fisher Scientific) was used for cDNA synthesis. Gene expression was measured with SYBR Green PCR Master Mix, 10 ng/µL cDNA and respective primers listed in Table S1. Quantstudio 6 Flex Real‐Time PCR System was used to run the experiment. ΔΔCT values were calculated by comparing the treated group with its respective untreated group.

### 4.6 Protein lysates and Western blot

Preparation of protein lysates and Western blot analyses were performed as previously described.^21^ Briefly, cell pellets were first washed with cold phosphate buffered saline (PBS) fetal bovine serum, lysed in RIPA buffer (Sigma) with protease inhibitor cocktail for 30 min on ice and finally centrifuged ≥15 000×*g* for 30 min at 4°C to collect the supernatant as total soluble protein lysate. For muscle tissue (quadriceps), 30 mg of frozen tissue was first homogenised with 30–40 strokes in RIPA using a Dounce homogeniser (Wheaton Dounce Tissue Grinder, Catalog #357538), followed by shearing the tissue by passing through a 25‐gauge syringe. For further lysis, the homogenate was rotated at 4°C for ∼2 h, centrifuged at >20 000×*g* at 4°C for 30 min, and the supernatant was collected as soluble muscle protein lysate. The lysates were quantified using BCA assay (Thermo Fisher Scientific). A total of 20 µg of protein lysate was used for Western blot and Trans‐Blot Turbo system (Bio‐Rad Laboratories) was used to electro‐transfer proteins to 0.45‐µm polyvinylidene difluoride (PVDF) membranes (Thermo Fisher Scientific) and immunoblotted with primary antibodies against glyceraldehyde 3 phosphate dehydrogenase (GAPDH) (1:2500; Abcam 9485), TDP‐43 (1:2500; Abcam 190963), LC3B (1:2000; Abcam 192890), SQSTM1/p62 (1:15 000; Abcam 56416), p‐TDP‐43 (1:2500; Cosmo Bio USA: CAC‐TIP‐PTD‐M01A), ubiquitin (1:1000; Abcam 7780) and VCP (1:20 000; Abcam 11433). Secondary antibodies used were either anti‐rabbit horseradish peroxidase (HRP) (1:5000) or goat anti‐mouse HRP (1:5000).

### 4.7 Immunohistochemistry

Immunohistochemical analyses were conducted on ASO‐treated SMPCs and myotubes differentiated from SMPCs. SMPC were either cultured in matrigel coated eight‐well chamber slides (Ibidi; IbiTreat 80826) for SMPC characterisation or in four‐well chamber slides (IbiTreat 80426) for myotube differentiation study and ASO treatment on SMPCs. After the specified time, the cells were fixed in 4% paraformaldehyde. The fixed SMPCs were permeabilised with 0.2% Triton‐X, blocked with Universal Block Buffer followed by incubation at 4°C overnight with primary antibodies for MF20 (1:100; DHSB), Pax7 (1:50; DSHB), SIX1 (1:100; Novus Biologicals NPB2‐52873), TDP‐43 (1:200), LC3B (1:1000), SQSTM1/p62 (1:200), ubiquitin (1:100; Santa Cruz sc‐8017), VCP (1:500), p‐TDP‐43 (1:2000) or galectin‐3 (1:300; Santa Cruz A3A12). The cells were then washed three times with PBS and incubated with the secondary antibodies donkey anti‐rabbit Cyanine Cy™3 (Jackson ImmunoResearch; 711 165 152); or donkey anti‐mouse Alexa Fluor™ 568 (Thermo Fisher Scientific; A10042; 1:500) at room temperature for 1 h, then washed and mounted with DAPI‐containing mounting media (Vectashield; Vector Laboratories, H‐1200‐10). All images were acquired with a Zeiss LSM 900 confocal microscope (Carl Zeiss). The mean fluorescence intensity was evaluated with ImageJ. Three images were acquired for each group, and at least 30 cells were analysed.

### 4.8 In vivo treatment of the VCP A232E mice with ASOs

Control non‐targeting ASO and VCP ASOs were reconstituted in PBS to a final concentration of 10 mg/mL. VCP A232E mice were injected subcutaneously with control ASO, or VCP ASO at 50 mg/kg weekly. For the safety and tolerability experiment (Figure S3), 10‐month‐old VCP A232E were dosed with control ASO, or VCP ASO at 50 mg/kg weekly for 3 months and compared with WT mice. Mice were regularly monitored for weight loss, alertness and activity. Weight loss of ≥15% compared with baseline was considered significant.

Serum was obtained from mice after 3 months of treatment with either control ASO, or VCP ASO, and used to measure ALT, AST and CK. Organ weights (liver, kidneys, spleen) were collected and analysed relative to body weight. For chronic treatment with VCP ASO to assess safety and efficacy, 6‐month‐old male VCP A232E mice were injected weekly with control ASO (*n* = 11) or VCP ASO (*n* = 11) for 3 months and assessed for motor function and compared with WT mice (*n* = 16). After 3 months of treatment, quadriceps muscle was collected for qPCR, Western blot and histological assessment using H&E staining. In this study, we focused on the quadriceps muscle, which exhibits prominent pathology in both VCP patients and VCP disease mouse models.^49^, ^64^, ^65^ As one of the most affected fast twitch predominant muscles, it provides a sensitive tissue for detecting treatment responses.

## 5 MUSCLE STRENGTH MEASUREMENT

### 5.1 Rotarod accelerating speed test

The Rotarod test was performed using a five lane Rotarod system (Med Associates Inc.) to investigate motor coordination and learning skills by measuring the ability of the mouse to stay and run on the accelerated rod. Mice are trained to walk on a rotating rod and then assessed for their ability to maintain balance as the rod accelerates from 4 to 40 RPM for a maximum time of 5 min. The test is conducted over 3 consecutive days. Each day, the mice are assessed for three trials with a recovery phase of 1–2 min between trials. As soon as a mouse falls off the rod or starts to rotate with the Rotarod without running, the timer is stopped. The latency time to fall from the rod in seconds is recorded and average from three trials and the 3‐day average is used for the analysis.

### 5.2 Grip strength test

The grip strength test measures the muscle strength of the forelimbs in rodents. Grip strength is measured using a commercially available Grip Strength Meter (TSE Systems GmbH, Berlin, Germany). Each mouse is held by the base of the tail above a wire grid and gently lowered down until its front paws grasp the grid. The animal is then brought to an almost horizontal position and pulled back gently but steadily until the grip is released. Five trials testing with 1‐min intervals between trials are performed per animal. The force measurements are recorded from the five trials and averaged. The average of the 3 days of testing is used for the analysis.^21^

### 5.3 Inverted screen test

Inverted screen testing is used to assess the muscle strength of all limbs in mice. No prior training is required since the mouse has a natural motivation to hang on to the screen to avoid falling.^3^ Each mouse is placed on the metal wire screen for ∼1 min to allow it to adjust to the new environment. The mouse is then moved to the centre of the wire screen. The screen is flipped quickly, but gently, so the mouse is hanging upside down. The time during which the mouse is suspended upside down on the wire screen before falling is measured. The maximum time for a single trial is set at 300 s. The mouse is allowed to rest for ∼1 min before placing it on the wire again. The test is repeated for two additional trials, unless the mouse hangs on for 300 s (i.e., if the mouse hangs on for 210 s in trial 1 and 300 s in trial 2, then trial 3 was not performed). The hanging time in seconds is recorded, and the average of the three trials of testing is used for analysis.

### 5.4 Hematoxylin and Eosin (H&E) staining

Quadricep muscles from mice are harvested, flash frozen in 2‐methyl butane (Sigma) and embedded in optimal cutting temperature (OCT) compound. Tissue sections of 8 µm thickness are cut using a CryoStar NX50 (Epredia) microtome and subsequently stained with H&E stain following standard protocols. All images are acquired with BZ‐X800 microscope (Keyence)

### 5.5 Statistical analysis

Statistical analysis was performed using GraphPad Prism 9.1.2 software (GraphPad Software, Boston, MA). Data are presented as mean ± SEM. Multiple unpaired *t*‐tests were used for qPCR, WB densitometry quantification and immunofluorescence image analyses of VCP R155H patient in comparison with WT iPSC‐derived SMPCs. For WB analysis of WT and VCP R155H iPSC‐derived SMPCs treated with and without VCP ASO and with bafilomycin, two‐way ANOVA followed by Šídák's multiple comparisons test was used. One‐way ANOVA test followed by Dunnett's multiple comparisons test was used for qPCR and WB analyses in WT and VCP R155H iPSC‐derived SMPCs treated with different doses of VCP ASO. Two‐way repeated measures ANOVA followed by Dunnett's multiple comparisons test was used for motor testing data analysis using Rotarod, grip strength, inverted screen tests and for body weight analysis. One‐way ANOVA test followed by Dunnett's multiple comparisons test was used for qPCR and WB densitometry quantification, and organ weight analysis in treated A232E compared with WT mice and unpaired *t*‐test was used for central nuclei quantification. For blood toxicology and liver enzyme analyses, one‐way ANOVA followed by Fisher's least significant difference test was used. AUC analysis was performed on motor performance longitudinal data. Group‐level comparisons of AUC values were conducted using one‐way ANOVA followed by Dunnett's multiple comparisons test to evaluate treatment effects relative to control groups. The level of statistical significance was set at *p = .05, **p = .01, ***p = .001, ****p = .0001. The sample size for in vivo mouse treatment was determined based on expected changes in muscle strength over time (baseline, 3, 6 and 9 months) between treatment and control groups. Based on prior literature,^21^ we assumed a 15‐s change in latency to fall on the rotarod test for mutant control mice, with a standard deviation of 10 s. To detect a 10‐s difference between treatment and control groups with 80% power and a significance level of 0.0125 (adjusted for multiple timepoints), a sample size of 12 mice per group was calculated.

## AUTHOR CONTRIBUTIONS

*Writing—original draft, investigation, data analysis and conceptualisation*: Pallabi Pal, Lan Weiss and Cheng Cheng. *Investigation, formal analysis and critical review*: Alyaa Shmara, Victoria Boock, Danae Bosch, Marwan Youssef, Yasamin Fazeli and Megan Afetian. *Investigation, data analysis and conceptualisation*: Olga G. Jaime and Michael R. Hicks. *Design of ASOs, investigation, formal analysis, writing and critical review*: Michele Carrer, Tamar R. Grossman and Paymaan Jafar‐nejad. *Writing—review and editing, writing, supervision, resources, project administration and data curation*: Virginia Kimonis.

## CONFLICT OF INTEREST STATEMENT

Michele Carrer, Megan Afetian, Paymaan Jafar‐nejad and Tamar Grossman are current or former paid employees of Ionis Pharmaceuticals.

## ETHICS STATEMENT

All animal experiments were conducted in accordance with the Institutional Animal Care and Use Committee at the University of California, Irvine (UCI), under protocol IACUC AUP 25‐046. The SMPCs were made under hSCRO protocol: 2009‐1005. Fibroblasts obtained from Coriell were used to generate the iPSCS.

## Supporting information



Supporting Information

Supporting Information

Supporting Information

Supporting Information

Supporting Information

## Data Availability

The data that support the findings of this study are available from the corresponding author upon reasonable request.
