# Supplementary figures and images for: Antisense oligonucleotides targeting valosin‐containing protein ameliorate muscle pathology and molecular defects in cell and mouse models of multisystem proteinopathy
Source: Clin Transl Med. 2025 Dec 8;15(12):e70530. doi: 10.1002/ctm2.70530 (PMC12683293; doi:10.1002/ctm2.70530)

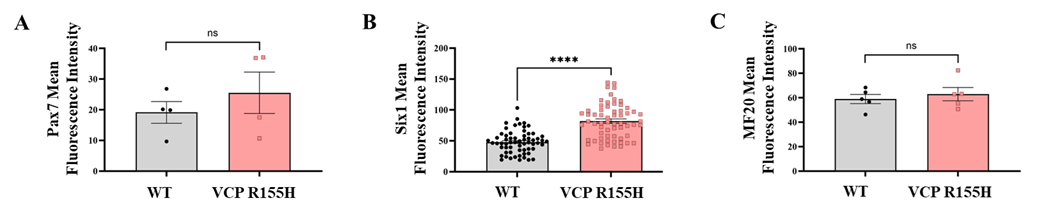

Supplement: Supplementary file 1 — Supporting Information [file CTM2-15-e70530-s004.tif]

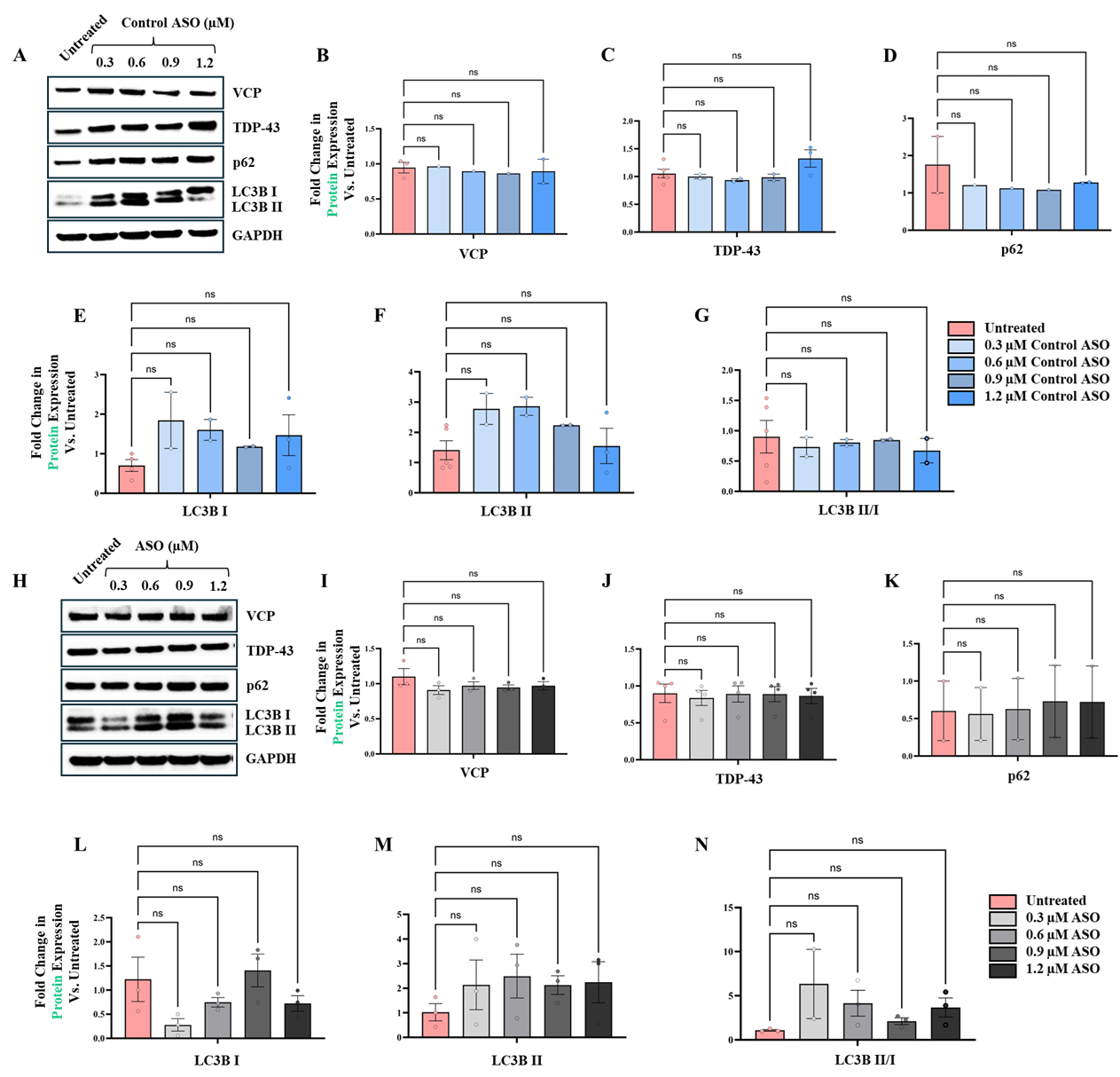

Supplement: Supplementary file 2 — Supporting Information [file CTM2-15-e70530-s002.tif]

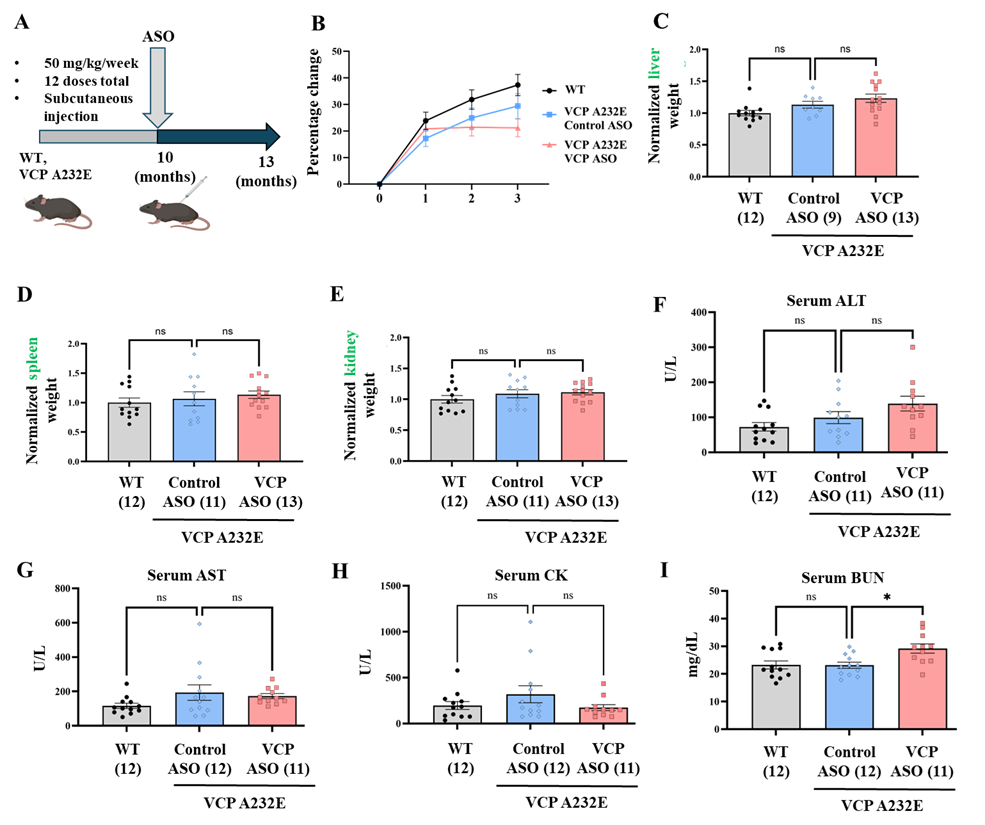

Supplement: Supplementary file 3 — Supporting Information [file CTM2-15-e70530-s001.tif]

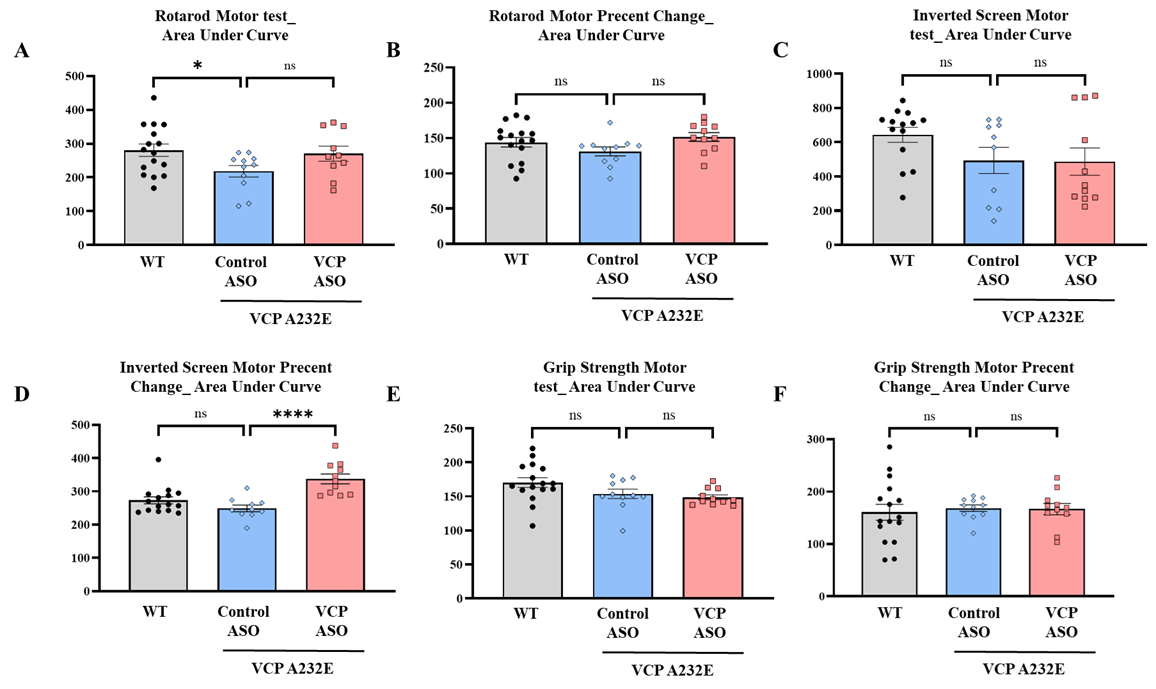

Supplement: Supplementary file 4 — Supporting Information [file CTM2-15-e70530-s003.tif]
