# Supplementary material for: Antisense oligonucleotides targeting valosin‐containing protein ameliorate muscle pathology and molecular defects in cell and mouse models of multisystem proteinopathy
Source: Clin Transl Med. 2025 Dec 8;15(12):e70530. doi: 10.1002/ctm2.70530 (PMC12683293; doi:10.1002/ctm2.70530)
Supplement: Supplementary file 5 — Supporting Information [file CTM2-15-e70530-s005.docx]

| **Supplementary Table 1: Sequences of gene-specific primers used for real-time PCR.** | | | |
| --- | --- | --- | --- |
| Species | Genes | Forward | Reverse |
| Human/Mouse | GAPDH | 5′-CTCTGCTCCTCCTGTTCGAG | 5′-TGGTGTCTGAGCGATGTGG |
| Human | SQSTM1/p62 | 5′-TGTGTAGCGTCTGCGAGGGAAA | 5′-AGTGTCCGTGTTTCACCTTCCG |
|  | LC3B | 5′-CAGCATCCAACCCAAAATCCC | 5′-GTTGACATGGTCAGGTACAAG |
|  | TDP 43 | 5′-GATGGACGATGGTGTGACTGCA | 5′-AAGAACTCCCGCAGCTCATCCT |
|  | VCP | 5′-GAGGAATCCTGCTTTACGGACC | 5′-GGCTTTACGAAGGTTGCTCTCAG |
| Mouse | SQSTM1/p62 | 5′-CTTACGGGTCCTTTTCCCAAC | 5′-TCCTCCTTGCCCAGAAGATAG |
|  | LC3B | 5′-CGTCCTGGACAAGACCAAGT | 5′-CCATTCACCAGGAGGAAGAA |
|  | TDP 43 | 5′- TTCATTCCCAAACCATTCAG | 5′-AGATGAACTGGATTACCACC |
|  | VCP (WT) | 5′-CACAGTGGAAGGCATCACTGG | 5′-AAGGGCTGGGATCTGTCTCT |
|  | VCP (A232E) | 5′-CAACGTGCTGGTTGTTGTG | 5′-AAGGACGATGCAAACAGCTT |
